# Clinical bracket failure rates between different bonding techniques: a systematic review and meta-analysis

**DOI:** 10.1093/ejo/cjac050

**Published:** 2022-10-12

**Authors:** Csaba Dudás, László Márk Czumbel, Szabolcs Kiss, Noémi Gede, Péter Hegyi, Krisztina Mártha, Gábor Varga

**Affiliations:** Doctoral School, George Emil Palade University of Medicine, Pharmacy, Science, and Technology, Târgu Mureș, Romania; Centre for Translational Medicine, Semmelweis University, Budapest, Hungary; Department of Oral Biology, Faculty of Dentistry, Semmelweis University, Budapest, Hungary; Doctoral School of Clinical Medicine, Faculty of Medicine, University of Szeged, Hungary; Institute for Translational Medicine, Szentágothai Research Centre, Medical School, University of Pécs, Hungary; Institute for Translational Medicine, Szentágothai Research Centre, Medical School, University of Pécs, Hungary; Centre for Translational Medicine, Semmelweis University, Budapest, Hungary; Institute for Translational Medicine, Szentágothai Research Centre, Medical School, University of Pécs, Hungary; Department of Orthodontics, Faculty of Dentistry, George Emil Palade University of Medicine, Pharmacy, Science, and Technology, Târgu Mureș, Romania; Centre for Translational Medicine, Semmelweis University, Budapest, Hungary; Department of Oral Biology, Faculty of Dentistry, Semmelweis University, Budapest, Hungary

## Abstract

**Background:**

Bracket failure increases the treatment time of orthodontic therapy and burdens patients with unnecessary costs, increased chair time, and possible new appointments.

**Objective:**

To compare the bond failures of different orthodontic materials based on the results of available clinical studies.

**Search methods:**

A systematic search of clinical trials was performed in the Cochrane, Embase, and Pubmed databases with no limitations. The list of investigated techniques contained conventional acid-etch primer (CM-AEP), self-etch primer (SEP), self-cure resin (SCR), and simple or resin-modified glass ionomer (RM-GIC) materials and procedures.

**Selection criteria:**

Clinical studies reporting the failure rate of bonded brackets after using direct adhesive techniques on buccal sites of healthy teeth were included.

**Data collection and analysis:**

Bracket failure rates from eligible studies were extracted by two authors independently. Risk ratios (RRs) were pooled using the random-effects model with DerSimonian–Laird estimation.

**Results:**

Thirty-four publications, involving 1221 patients, were included. Our meta-analysis revealed no significant difference in the risk of bracket failures between SEP and CM-AEP. After 6, 12, and 18 months of bonding, the values of RR were 1.04 [95% confidence interval (CI), 0.67–1.61], 1.37 (95% CI, 0.98–1.92), and 0.93 (95% CI, 0.72–1.20), respectively. At 18 months, bracket failure was 4.9 and 5.2% for SEP and CM-AEP, respectively. Heterogeneity was good or moderate (*I*^2^ < 42.2%). The results of RM-GIC at 12 months indicated a 57% lower risk of bracket failure using SCR as compared with RM-GIC (RR: 0.38; 95% CI, 0.24–0.61). At 18 months, bracket failures for SCR and RM-GIC were 15.8 and 36.6%, respectively (RR: 0.44; 95% CI, 0.37–0.52, *I*^2^ = 78.9%), demonstrating three to six times higher failure rate than in the case of etching primer applications.

**Limitations:**

A major limitation of the present work is that the included clinical trials, with no exceptions, showed variable levels of risk of bias. Another possible problem affecting the outcome is the difference between the clustering effects of the split mouth and the parallel group bracket allocation methods.

**Conclusions and implications:**

The results revealed no significant difference between SEP and CM-AEP up to 18 months after application. RM-GIC had much worse failure rates than acid-etching methods; additionally, the superiority of SCR over RM-GIC was evident, indicating strong clinical relevance.

**Registration:**

Prospero with CRD42020163362.

## Introduction

Since the introduction of orthodontic treatment using directly bonded brackets on enamel, which replaced the multiband type of fixed appliance, new technologies (e.g. involving the moisture and technique sensitivity of bonding materials) have followed the old methods. While the biggest challenges of legacy therapies were the time-consuming separation of teeth, the application of bands, and the closure of interproximal spaces, newly applied direct bonding has the disadvantage of possible failure during treatment (1). Additionally, accidental debonding of brackets increases treatment time and costs (2,3). Furthermore, the rebonding procedures may increase the risk of possible redundant damage to the enamel (4).

During the development of orthodontic bonding materials, developers need to keep in mind the different expectations of practitioners. The bond strength needs to combat the traction of archwires and the forces of occlusion and mastication (5). On the other hand, the removal of brackets at the end of treatment needs to be easy, without side-effects (6). Adequate curing time and simplicity of technique are still important requirements (1).

The introduction of visible-light-cured composite materials resulted in a breakthrough in modern bracket bonding. They shortened the polymerization time, and the clinician is able to directly induce photopolymerization in contrast to the catalyst- or liquid activator-based self-cure techniques. The aspirations to further reduce chair time initiated the appearance of adhesive precoated brackets which do not require manual application of a composite to the bracket base (7). The combination of etching and priming with a self-etching primer (SEP) simplified bracket bonding and excluded the acid-etch stage of the conventional process of acid-etching primers (CM-AEP) (8). Studies that evaluated the chair time length found it significantly faster to use SEP compared with CM-AEP (9–11).

An alternative material for bracket bonding is glass ionomer. After its introduction by Wilson and Kent in 1972 (12), investigators started to study the orthodontic application of this material. Despite adverse properties such as fluoride release and adhesion to both enamel and metals, the introduction of a resin-modified light-curable version of glass ionomer broadened its indication area (13). Due to the contradictory results, it is used in orthodontics mainly for banding and bonding on wet surfaces (1).

Apparently, there is a high number of randomized and non-randomized clinical trials investigating bracket failure of orthodontic bonding materials, but most of them have relatively small sample sizes, and their outcomes were quite variable and heterogeneous. To combat the weaknesses of individual clinical studies, three meta-analyses attempted to extend our knowledge of evidence-based orthodontics (14–16) by comparing etching based techniques (14,16) in one hand, and resin and glass ionomer-based methods on the other hand (15,17) in separate meta-analyses. Unfortunately, those already published meta-analyses did not include all available studies on bracket failure and yielded contradictory results. In addition, they did not investigate the time dependency of bracket failure either, even though this is a very important aspect of potential failures since bonded brackets are intended to remain in place in the patient’s mouth for a long period of time.

Therefore, the objective of the present meta-analysis and systematic review was to quantitatively and qualitatively analyse the time dependence of bracket failure, synthesizing the results of all available clinical studies to further increase the precision and statistical power of presently available evidence. According to our null hypothesis, there were no significant differences in the bracket failure rates of different orthodontic bonding techniques. We statistically compared the results of the SEP technique with the CM-AEP technique after 6, 12, and more than 18 months of orthodontic treatment, and also of self-cure resin (SCR) with RM-GIC applications after 12- and 18-month follow-up periods.

## Methods

### Eligibility criteria review

This meta-analysis was reported according to the PRISMA 2020 statement, an updated guideline for reporting systematic reviews (18). The PRISMA checklist for our work is available in Supplementary Tables 1 and 2. It was registered in Prospero (International prospective register of systematic reviews) at the 01/06/2020 study selection with the ID number CRD42020163362.

Due to the large amount of data, the list of outcomes originally registered in Prospero was narrowed to bracket failure, while bonding time, Adhesive Remnant Index (ARI), and demineralization have not been processed statistically in this paper. Additionally, subgroup analyses were considered when the number of included studies permitted.

### Search strategy

A systematic search for articles was made in the MEDLINE (via PubMed), Embase, and Cochrane Central Register of Controlled Trials (CENTRAL) electronic databases with the following search string: [(glass ionomer) OR (dual cure) OR (self cure) OR (SEP OR self etch OR self etching) OR (acid etch OR two-step etch OR bonding agent OR primer)] AND (bracket OR braces OR brackets). Searches contained results up to the 22nd of October 2021. No language or study design restrictions were applied during the search process. Supplementary Table 3 contains the detailed search strategy used for each database.

### Eligibility and selection criteria

According to the PICO format, the following clinical question was formulated: Are there differences in bracket failure rate with one-step/two-step bonding procedures and applications of glass ionomer cements? The framework contained the following components: Population—patients with intact teeth, without prosthodontic restorations, who needed to undergo an orthodontic direct bonding procedure; Intervention and Comparison—comparison of multiple procedures, such as self-etching or conventional etching methods, light-curable or self-cured composite, glass ionomer or resin-modified glass ionomer materials, uncoated or adhesive precoated brackets; and Outcome—the failure rate of brackets.

Inclusion and exclusion criteria: clinical studies [randomized controlled trials (RCTs), non-randomized controlled trials (NRCTs), and controlled before–after studies] which analysed the failure rate of bonded brackets after using different direct adhesive techniques (including both precoated and operator coated brackets) on buccal sites of healthy teeth met the eligibility criteria for inclusion. Both split-mouth (S-M) and parallel-group (P-G) study designs have been accepted. But in this case we formed subgroups of the investigations using the two study designs. Publications that applied banded attachments, indirect orthodontic bonding techniques, or tested in vitro shear bond strength fulfilled the exclusion criteria.

Search results were handled with the help of Mendeley reference management software. Duplicated references were removed, and the remaining records were examined individually by two authors (CD and LMC). The primary method of selection was based on a check of the publication’s title and abstract, followed by full-text assessments of studies that passed the previous stage. Disagreements between authors were resolved either by discussion or by consultation with a third author (GV).

### Data extraction

Data extraction was performed by the two authors (CD and LMC) using a preconstructed Excel table. The following pieces of information were collected: name of first author, publication year, study design (S-M/P-G), follow-up period, number of participants, gender distribution, age, bracket type, involved techniques of intervention and comparison, and outcomes (bracket failure, bonding time, ARI). In cases of disagreement, the reviewers relied on the opinion of a third author (GV).

### Risk-of-bias assessment and certainty of evidence

Based on the recommendations of the Cochrane Handbook for Systematic Reviews of Interventions, bias evaluations were performed using the revised Cochrane risk-of-bias tool (RoB 2) for randomized trials (19) and the Risk of Bias in Non-randomized Studies—of Interventions (ROBINS-I) for non-randomized trials (20). The eligible randomized studies were rated as either 1. low risk of bias, 2. some concerns, or 3. high risk of bias, across the following domains: randomization process, deviations from intended interventions, missing outcome data, measurement of the outcome, and selection of the reported results. Authors rated the overall bias as low risk if more than three categories were rated as having a low risk of bias and the remaining two had some concerns; in other cases of concern variations without marks of high risk, publications were marked as having some concerns in the overall evaluation. Non-randomized studies were assessed on bias due to confounding, selection, classification on interventions, deviations from intended interventions, missing data, outcome measurement, and reported results.

The evaluation with both methods was undertaken independently by two reviewers (CD and LMC) and possible discrepancies were resolved by discussion or the intervention of third author (GV).

We used the Grading of Recommendations Assessment, Development, and Evaluation approach to assessing the certainty of evidence (21). The evaluation of each assessment criteria for each outcome and comparison was made by the above mentioned reviewers with third-party arbitration.

### Statistical analysis

We provided summaries of intervention effects for each study by calculating risk ratios (RRs) for our dichotomous outcomes using the Stata 11 SE software (StataCorp LLC, College Station, Texas, USA). RRs were pooled using the random-effects model with DerSimonian–Laird estimation and displayed on forest plots. Summary RR estimation, *P* value, and 95% confidence interval (CI) have been calculated. Statistical heterogeneity was analysed by calculating prediction intervals, *I*^2^ statistics and the *χ*^2^ tests to acquire probability values; *P* < 0.1 was defined to indicate significant *I*^2^ values. The minimum number of studies for performing a meta-analysis was two. As the number of eligible studies was high enough, we were able to test the presence of publication bias by creating funnel plots and performing Egger’s tests. Subgroup analyses for different study designs (RCTs and NRCTs) and bracket allocation methods (S-M and P-G) were also performed. The only limitation to create subgroups was the presence of at least two studies in each statistical group.

## Results

### Systematic search and selection

**Figure 1.**
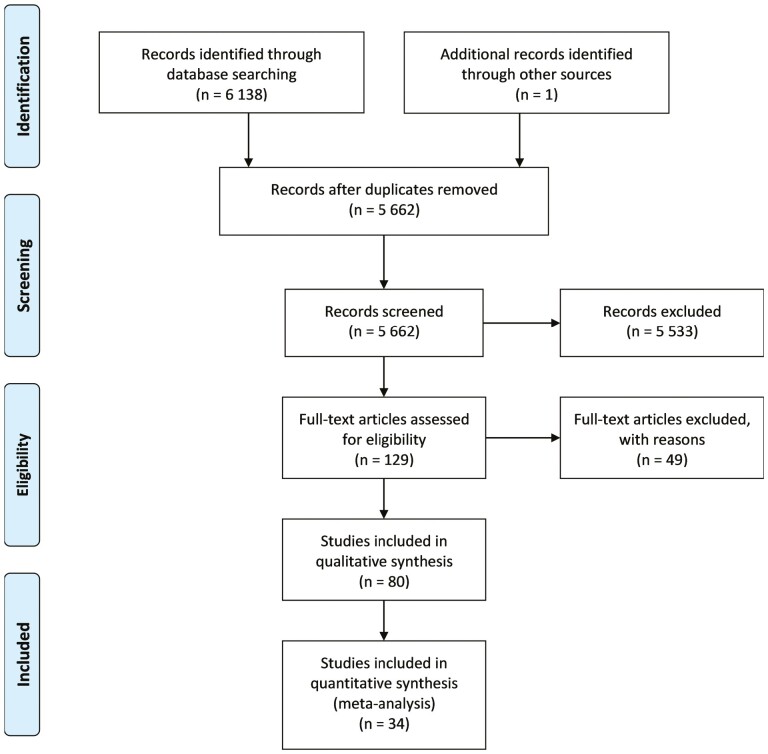
PRISMA flow diagram of the study selection process.

A total of 6138 records were identified through database searching. After removing duplicates, 5662 articles were screened. During the screening process, 5533 articles were excluded and only 129 were considered eligible for full-text assessment. Of these publications and after exclusions, 80 studies were included in the qualitative and 34 in the quantitative syntheses (8–11,22–50). The selection process is shown in Figure 1. Reasons for the exclusion of individual studies in the full-text assessment are detailed in Supplementary Table 4.

### Characteristics of the studies included

#### Description of excluded studies

The studies which did not meet the expectations of inclusion criteria were definitively excluded. The presence of different examined outcomes, inadequate study design, or indirect bonding technique was the common reasons for exclusion (Supplementary Table 4). A number of studies were included in the qualitative but not in the quantitative analysis as in several cases there was an insufficient number of studies having the same outcome to perform a meta-analysis on the records. These investigations were only presented in the part of systematic review of this paper (Supplementary Document 1).

#### Description of included studies

The majority of included studies in the quantitative synthesis were RCT. Based on the bonding technique and the materials used, they could be organized into distinct groups. Articles that investigated the bond failure involving the SEP or CM-AEP techniques with light-curable composite were classified into 6-, 12-, and over 18-month groups. The results of studies of SCR compared with glass ionomer cements (GICs) were categorized as per their follow-up periods into 12- and 18-month groups. In these groups, we found the survival results of 6599 brackets bonded with SEP, 6570 with CM-AEP, 4029 with SCR, and 4123 cemented with GIC. The characteristics and detailed descriptions of the included studies are shown in Table 1.

**Table 1.** Summary of study characteristics.

Study	Design	Treatment allocation	No. of patients	Age in years (mean and range)	Gender distribution (female%)	Follow-up period(in months)	Intervention/comparison	Outcome
Aljubouri *et al.* (2004)	Split-mouth	SEP versus CM-AEP	51	n.d. [n.d.]	68%	6/12	SEP/CM-AEP	Bracket failure, bonding time
Ousehal *et al.* (2016)	Split-mouth	SEP versus CM-AEP	100	n.d. [n.d.]	80%	6	SEP/CM-AEP	Bracket failure
Elekdag-Turk *et al.* (2008) (1)	Split-mouth	SEP versus CM-AEP	37	16.5 [n.d.]	62%	6	SEP/CM-AEP	Bracket failure, bonding time, Adhesive Remnant Index
Elekdag-Turk *et al.* (2008) (2)	Split-mouth	SEP versus CM-AEP	39	16.5 [n.d.]	79%	12	SEP/CM-AEP	Bracket failure, Adhesive Remnant Index
Dos Santos *et al.* (2006)	Split-mouth	SEP versus CM-AEP	30	n.d. [12–18]	50%	6	SEP/CM-AEP	Bracket failure
Romano *et al.* (2012)	Split-mouth	SEP versus CM-AEP	20	n.d. [10.5–15.1]	65%	6	SEP/CM-AEP/SCR	Bracket failure
Cal-Neto *et al.* (2005)	Split-mouth	SEP versus CM-AEP	15	n.d.	n.d.	6	SEP/CM-AEP	Bracket failure
Asgari *et al.* (2002)	Split-mouth	SEP versus CM-AEP	20	n.d.	n.d.	6	SEP/CM-AEP	Bracket failure
Ireland *et al.* (2003)	Split-mouth	SEP versus CM-AEP	20	n.d.	n.d.	6	SEP/CM-AEP	Bracket failure
Manning *et al.* (2006)	Split-mouth	SEP versus CM-AEP	34	13.7 [n.d.]	67%	6/12	SEP/CM-AEP	Bracket failure
Cal-Neto *et al.* (2009)	Parallel-group	SEP versus CM-AEP	28	14.9 [n.d.]	60%	12	SEP/CM-AEP	Bracket failure
Murfitt *et al.* (2006)	Split-mouth	SEP versus CM-AEP	39	14.4 [n.d.]	66%	12	SEP/CM-AEP	Bracket failure, Adhesive Remnant Index
Dandachli *et al.* (2015)	Split-mouth	SEP versus CM-AEP	46	n.d. [15–20]	71%	12	SEP/CM-AEP/RM-GIC	Bracket failure, Adhesive Remnant Index
Atik *et al.* (2018)	Parallel-group	SEP versus CM-AEP	63	15.2 [n.d.]	73%	12	SEP/CM-AEP	Bracket failure, Adhesive Remnant Index
Manning *et al.* (2006)	Parallel-group	SEP versus CM-AEP	34	13.7 [n.d.]	67%	12	SEP/CM-AEP	Bracket failure
Pandis *et al.* (2006)	Split-mouth	SEP versus CM-AEP	22	13 [12–14]	59%	12	SEP/CM-AEP	Bracket failure
Dominguez *et al.* (2013)	Split-mouth	SEP versus CM-AEP	24	n.d. [n.d.]	n.d.	18	SEP/CM-AEP	Bracket failure
Sam *et al.* (2012)	Split-mouth	SEP versus CM-AEP	80	16 [13–33]	58%	18	SEP/CM-AEP	Bracket failure
Ozer *et al.* (2014)	Split-mouth	SEP versus CM-AEP	57	16 [n.d.]	68%	18	SEP/CM-AEP	Bracket failure, Adhesive Remnant Index
Reis *et al.* (2008)	Split-mouth	SEP versus CM-AEP	30	n.d. [12–18]	50%	18	SEP/CM-AEP	Bracket failure
Banks *et al.* (2007)	Parallel-group	SEP versus CM-AEP	60	n.d. [n.d.]	61%	18	SEP/CM-AEP	Bracket failure, bonding time, Adhesive Remnant Index
Gaworski *et al.* (1999)	Split-mouth	SCR versus RMGIC	16	n.d. [n.d.]	n.d.	12	SCR/RM-GIC	Bracket failure, Decalcification
Cacciafesta *et al.* (1999)	Split-mouth	SCR versus RMGIC	38	n.d. [n.d.]	n.d.	12	SCR/RM-GIC	Bracket failure
Fricker *et al.* (1992)	Split-mouth	SCR versus RMGIC	10	n.d. [n.d.]	n.d.	12	SCR/RM-GIC	Bracket failure
Fricker *et al.* (1994)	Split-mouth	SCR versus RMGIC	10	n.d. [n.d.]	n.d.	12	SCR/RM-GIC	Bracket failure
Fricker *et al.* (1998)	Split-mouth	SCR versus RMGIC	10	n.d. [n.d.]	n.d.	12	SCR/RM-GIC	Bracket failure
Hegarty *et al.* (2002)	Split-mouth	SCR versus RMGIC	61	14.2 [n.d.]	75%	12	SCR/RM-GIC	Bracket failure
Miguel *et al.* (1995)	Split-mouth	SCR versus RMGIC	16	12 [10–15]	50%	12	SCR/RM-GIC	Bracket failure
Millett *et al.* (1999)	Split-mouth	SCR versus RMGIC	40	13.4 [n.d.]	57%	12	SCR/RM-GIC	Bracket failure, decalcification
Fowler *et al.* (1998)	Parallel-group	SCR versus RMGIC	50	n.d. [n.d.]	n.d.	12	SCR/RM-GIC	Bracket failure
Ireland *et al.* (2002)	Split-mouth	SCR versus RMGIC	60	n.d. [n.d.]	n.d.	18	SCR/RM-GIC	Bracket failure
Miller *et al.* (1996)	Parallel-group	SCR versus RMGIC	17	n.d. [n.d.]	35%	18	SCR/RM-GIC	Bracket failure
Norevall *et al.* (1996)	Parallel-group	SCR versus RMGIC	30	n.d. [n.d.]	56%	18	SCR/RM-GIC	Bracket failure, adhesive remnant, clean-up time
Oliveira *et al.* (2004)	Split-mouth	SCR versus RMGIC	14	n.d. [n.d.]	71%	24	SCR/RM-GIC	Bracket failure

CM-AEP, conventional acid-etch primer; n.d., no data; RM-GIC, resin-modified glass ionomer; SCR, self-cure resin; SEP, self-etch primer.

Patients selected for the trials represented both sexes, ranging from 10 to 21 years of age. All participants involved in the clinical trials needed orthodontic treatment. The presence of direct and indirect restorations on buccal surfaces, congenital enamel defects, and caries constituted exclusion criteria. All authors ignored the categorization of gender, age, and race-related properties. In all studies except for three trials, the authors recognized or published no contradictory information about the inclusion of all types of orthodontic malocclusion cases. One study involved only non-extraction Class I malocclusion cases (24), another excluded Class III cases with non-balanced extractions (11), and the third did not consider patients with Class III or openbite cases (45).

The allocation of brackets was predominantly based on S-M design. In principle, quadrants assigned to each adhesive or bonding technique were consequently alternated in the mouth. Thus, the same participant was involved equally in the intervention and in the comparison group. In a few exceptions, the patients were distributed in one of the groups, receiving brackets with the same protocol in all quadrants (P-G or whole-mouth design) (8,39,40).

The installation of the orthodontic appliances started with a pumice-based mechanical cleaning of the teeth. Ireland and Sherriff also examined the effect of leaving on the initial prophylaxis on the failure rate of brackets (44). Other authors did not provide sufficient data on this topic (38,40,48).

The application of acid to the enamel of teeth was the next phase of tooth preparation before bracket bonding. In the case of CM-AEP, the vast majority of studies used 37% phosphoric acid. Only one clinical trial reported the use of 30% phosphoric acid (39), while some other authors did not mention the concentration (34,38,40,46,51). The described conditioning time varied in different articles from 15, 20, 30 to 60 seconds. Some papers reported rubbing the enamel surface for 3, 3–5, 5, 10–15, or 20 seconds, and then applying a gentle air jet. In some studies (33,34,36,40), the application of GIC preceded the application of polyacrylic conditioner for 10 seconds, and one author reported the use of 37% phosphoric acid for 60 seconds (37). In the remainder of the clinical trials, the protocol did not include or mention acid treatment before the use of the GIC (24,31,32,38,39,42,44).

Except for one-stage SEP and GIC procedures, the next step during the orthodontic process was the application of different types of primers. The placement of an adhesive on the base of new, uncoated brackets was followed by their direct positioning by the clinician on the labial tooth surface. The light-curing time of resin-based materials in each interspace was predominantly 20 seconds. One study reported 10 seconds (48) and some authors also applied the light from the gingival, incisal, or oral directions, thus increasing the total exposure time per tooth (34,46).

### Results of individual studies and synthesis of results

#### No difference in bracket failure between SEP and CM-AEP techniques after 6, 12, and more than 18 months

**Figure 2.**
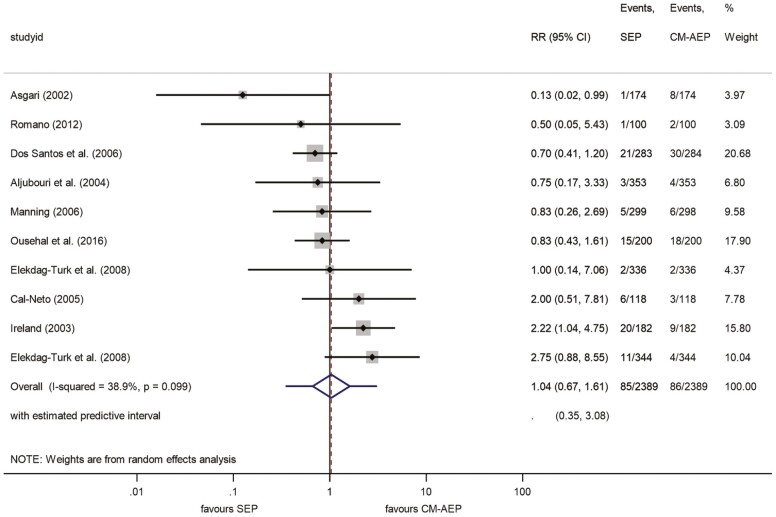
Forest plot analysis of bracket failures between SEP and CM-AEP after 6 months. CM-AEP, conventional acid-etch primer; SEP, self-etch primer.

The results were obtained from 10 studies which examined the failure rate of bonded brackets with the SEP and CM-AEP techniques, showing the evolution of performance between them after a 6-month period (9,11,22,41,45,46,48–50,52). Our meta-analysis highlighted that 3.6% of brackets failed in the SEP and 3.6% failed in the CM-AEP group. This difference was non-significant (RR: 1.04, 95% CI, 0.67–1.61) (Figure 2). The value of *I*^2^ = 38.9% indicated a negligible heterogeneity level. On the other hand, the predictive interval was found to be in the range 0.35–3.08. This means that the ‘risk’ ratio for bracket failure is estimated to fall into the range of 0.35–3.08. The leave-one-out analysis resulted in no significant changes (Supplementary Figure 1), suggesting that leaving out one of the studies did not influence the final results. The Egger’s test suggested no small-study effect (*P* = 0.890).

**Figure 3.**
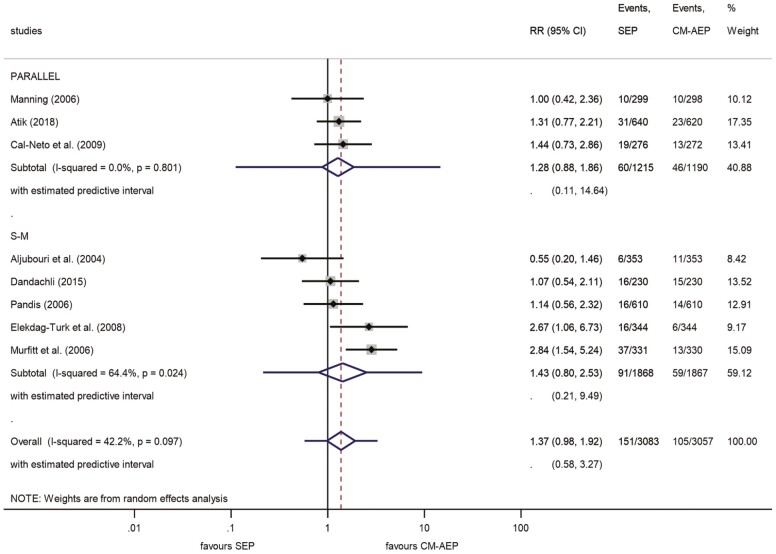
Forest plot analysis of bracket failures between SEP and CM-AEP after 12 months. Subgroup analysis (S-M–parallel). CM-AEP, conventional acid-etch primer; SEP, self-etch primer; S-M, split-mouth.

**Figure 4.**
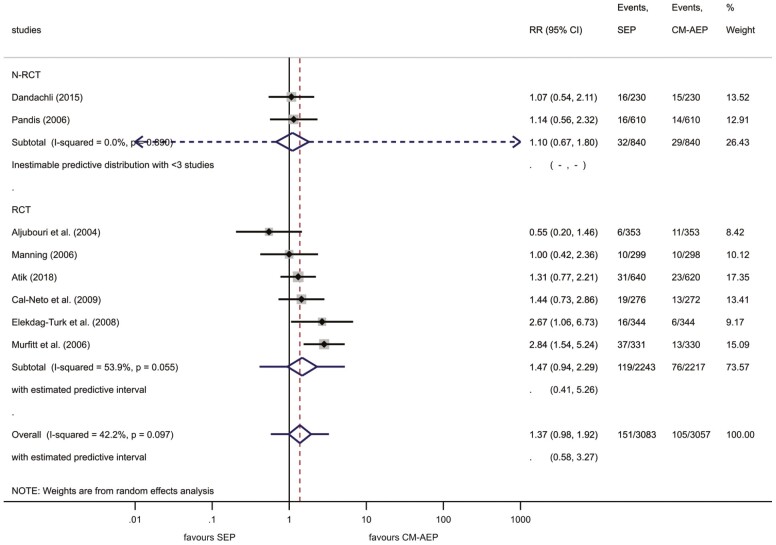
Forest plot analysis of bracket failures between SEP and CM-AEP after 12 months. Subgroup analysis (NRCT–RCT). CM-AEP, conventional acid-etch primer; NRCT, non-randomized controlled trial; RCT, randomized controlled trial; SEP, self-etch primer.

After 12 months of bonding, we calculated a 4.9% failure in the SEP compared with a 3.4% failure in the CM-AEP group. The RR for failure was 1.37 (95% CI, 0.98–1.92), showing a tendency to favour the CM-AEP method (8,9,22–26,45) (Figures 3 and 4). This indicated that the relative risk of bracket failure showed a tendency to become statistically significant at a moderate level of heterogeneity (overall *I*^2^ = 42.2%). The overall estimated predictive interval was 0.58–3.27. The leave-one-out analysis was significant regarding the study of Aljubouri *et al.* (9). When this study was excluded, the RR was 1.49 (95% CI, 1.09–2.03), reaching a significant difference level favouring CM-AEP treatment (Supplementary Figure 2). Egger’s test suggests no small-study effect (*P* = 0.472).

We also performed a subgroup analysis to see the difference of S-M and P-G designs separately on the outcome of 12 months studies in bracket failure (Figure 3). We observed greatly overlapping intervals with very similar means without showing any tendency of significant difference between the S-M (RR: 1.43; 95% CI, 0.80–2.53) and the P-G (RR: 1.28; 95% CI, 0.88–1.86). The relative heterogeneity was lower in the studies which used P-G allocation (*I*^2^ = 0.0%) in comparison with those preferred the S-M method (*I*^2^ = 64.4%). The predictive intervals were in the range 0.11–14.64 in the P-G allocation subgroup, and 0.21–9.49 in the S-M studies.

Subgroup analysis to compare the outcome of RCTs and NRCTs were also executed to see any differences in bracket failure between SEP and CM-AEP at 12 months (Figure 4). Again, not even a tendency for differences between NRCTs (RR: 1.10; 95% CI, 0.67–1.60) and RCTs (RR: 1.47; 95% CI, 0.94–2.29) could be detected. But the relative heterogeneity was lower in NRCTs (*I*^2^ = 0.0%) in comparison with RCTs (*I*^2^ = 53.9%). The predictive interval of NRCTs indicating the absolute heterogeneity could not be estimated due to the number low number of NRCTs. The predictive interval for RCTs was 0.41–5.26.

**Figure 5.**
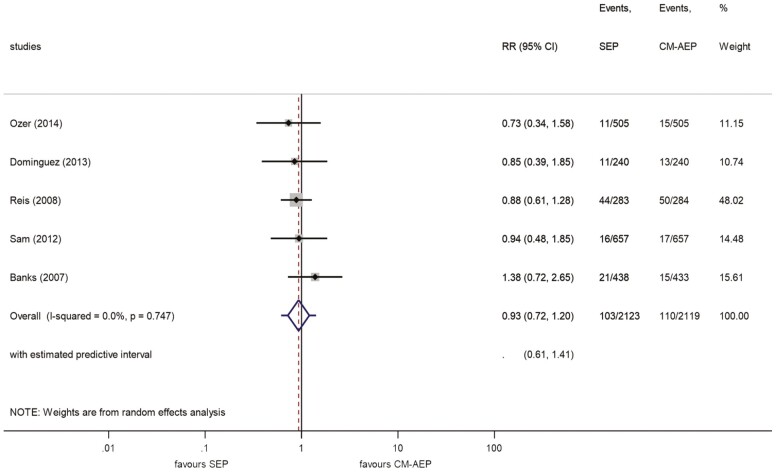
Forest plot analysis of bracket failures between SEP and CM-AEP after more than 18 months. CM-AEP, conventional acid-etch primer; SEP, self-etch primer.

Long-term bracket failure data were available in five studies (10,27–30). No significant difference between SEP and CM-AEP applications was found (RR: 0.93, 95% CI, 0.72–1.20) (Figure 5). The failure rates were 4.6% and 5.2% for SEP and CM-AEP, respectively. The inconsistency suggests small amounts of heterogeneity (*I*^2^ = 0.0%). The predictive interval was estimated to be in the range 0.61–1.41. The leave-one-out analysis yielded no significant results (Supplementary Figure 3).

#### Bracket failure was lower for SCR compared with RM-GIC at both 12 and 18 months

**Figure 6.**
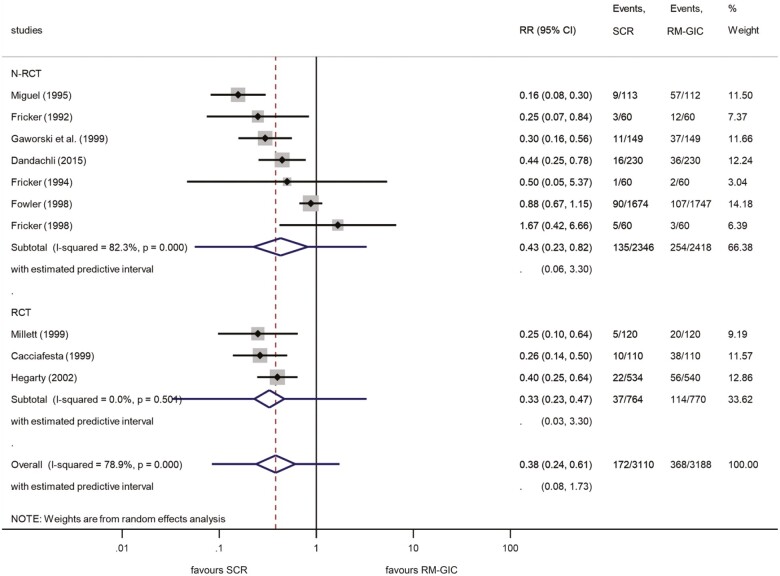
Forest plot analysis of bracket failures between SCR and RM-GIC after 12 months. Subgroup analysis (NRCT–RCT). NRCT, non-randomized controlled trial; RCT, randomized controlled trial; RM-GIC, resin-modified glass ionomer; SCR, self-cure resin.

The results of 10 studies (24,31–39) were synthesized by our meta-analysis comparing the differences in the bracket failure rate of SCR and GIC, and show the evolution of performance between them after 1 year. The failure rate was found to be 5.5% in the SCR group and 11.5% in the RM-GIC group. The calculated RR indicated a significant 62% lower risk of bracket failure if the bonding technique involved SCR against GIC (RR: 0.38; 95% CI, 0.24–0.61) (Figure 6). The high value of *I*^2^ (78.9%) suggested considerable heterogeneity between investigations. The leave-one-out analysis yielded no significant changes (Supplementary Figure 4), while Egger’s test presents no small-study effect (*P* = 0.129).

A subgroup analysis was also performed to compare RCT and NRCT designs on the outcome at 12 months bracket failure between SCR and GIC interventions. Once more, no tendency showing a significant difference between NRCTs (RR: 0.43; 95% CI, 0.23–0.82) and RCTs (RR: 0.33; 95% CI, 0.23–0.47) were detected (Figure 6). Importantly, the relative heterogeneity was lower for RCTs (*I*^2^ = 0.0%) in comparison with NRCTs (*I*^2^ = 82.3%). The predictive interval of NRCTs indicating the absolute heterogeneity was estimated to be in the range 0.06–3.30, while the interval for RCTs was 0.03–3.30.

**Figure 7.**
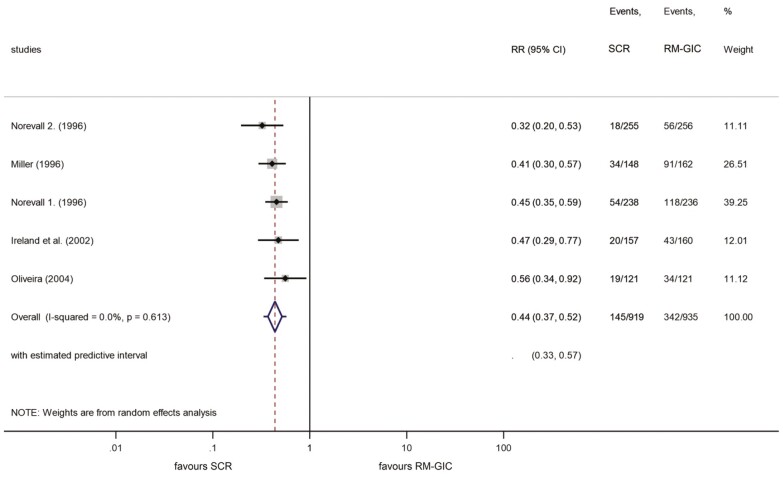
Forest plot analysis of bracket failures between SCR and RM-GIC after 18 months. RM-GIC, resin-modified glass ionomer; SCR, self-cure resin.

Four studies investigating the bond failure of SCRs and GIC after 18 months were available for data pooling (40,42–44). The results showed that in the SCR group 15.8% of the brackets failed, whereas in the RM-GIC group the figure was 36.6%. This great difference means the risk of bracket failure was 56% lower with the SCR technique compared with GIC application (RR: 0.44; 95% CI, 0.37–0.52) (Figure 7). The leave-one-out analysis resulted in no significant outcomes (Supplementary Figure 5). The predictive interval was in the range 0.33–0.57.

### Qualitative analysis

The findings of studies included in the qualitative part of our paper can be found in Supplementary Text 1.

### Risk-of-bias assessment and certainty of the evidence

The six randomized studies were rated to have a low risk-of-bias level (8–10,22,23,36). Although these studies presented some concerns of bias regarding blinding and selection, the randomization process was transparent and detailed. The remainder of the articles were judged to have more bias concerns. None of these studies were double blind as it was not possible to blind the operator to the bonding type during the studies. For this reason, no study could receive a higher score (Supplementary Table 5).

The non-randomized studies presented a low or moderate risk of bias. With one exception, all publications had some deficiency regarding participant selection (31). Using even and odd numbers at the level of teeth as a division method for intervention and control groups in one study was evaluated by reviewers as an attempt to reduce confounding variables. The rest of publications were evaluated to present a moderate level of confounding factors because of the lack of randomization and no intention to control patient inclusion by age, orthodontic or dental status. Results of the ROBINS-I tool for non-randomized studies can be found in Supplementary Table 6.

#### Certainty of evidence

According to our statistical results, the widest range of the predictive interval (0.11–14.64) was found when CM-AEP and SEP interventions were compared in the parallel oral application subgroup at 12 months. The widest overall predictive interval was found in the CM-AEP—SEP groups at 6 months (0.35–3.08).

We found that the level of certainty was very low (SEP versus CM-AEP 6, 12 months) and low (SEP versus CM-AEP 18 months). Due to the presence of NRCTs in all three mentioned groups, the certainty level was reduced. The decrease of final certainty level was proportional with heterogeneity. The highest level of *I*^2^ (*I*^2^ = 78.9%) and the proportion of NRCTs were observed in SCR-RMGIC groups. Detailed results of the GRADE assessment are found in Supplementary Table 7.

Publication bias was evaluated by funnel plot and Egger’s test. The funnel diagrams are shown in Supplementary Figure 6 for the CM-AEP—SEP comparison at 6 months, and in Supplementary Figure 7 at 12 months. Due to the insufficient number of studies no funnel plot was created for CM-AEP—SEP comparison over 18 months. Supplementary Figure 8 represents the funnel plot of the SCR—RM-GIC method after 12 months. The funnel plot of SEP and CM-AEP analysis after 6 months is a symmetrical scatter plot, with no signs of publication bias. Egger’s test (*P* = 0.890) indicates no small-study effect here. The missing dots (studies) on the funnel plot of SEP and CM-AEP after 12 months indicate that more studies are needed with increased sample size and statistical power. At 12 months Egger’s test (*P* = 0.472) indicates no small-study effect. The funnel plot of SCR and RM-GIC after 12 months (Supplementary Figure 8) presents slight asymmetry of the plot and indicates the presence of statistical heterogeneity, however Egger’s test (*P* = 0.129) suggests no small-study effect. Due to the limited number of studied, no funnel plot was created for SCR and RM-GIC comparison at 18 months.

### Sensitivity analysis

We conducted the leave-one-out sensitivity analysis for each calculated overall estimate. In each case none of the included records to significantly alter the overall estimate if each of the records is omitted; except for the study Aljubouri *et al.* (9). after 12 months of bonding. When omitting this study, a significant difference was received in bracket failure between the SEP and the CM-AEP after 12 months significantly changes the estimate of the analysis to RR: 1.49 95% CI (1.10–2.03). However, after careful examination of the records, we found no particular reason to exclude the study of Aljubouri *et al.* from our quantitative analysis. Supplementary Figures 1–5 are the graphical representations of the leave-one-out analysis for each overall estimate.

## Discussion

For decades, clinicians have constantly sought a low failure rate and single component orthodontic cements that do not require pre-treatment of the tooth surface (53). As a result, several in vitro studies and clinical trials investigated bracket survival using various materials and techniques, while attention gradually moved from GICs to adhesive materials (1). But available data are variable and still contradictory. To seek clear evidence, the meta-analyses must be performed to send clear messages to practitioners. Using this methodology, we increased the power to detect bracket failure rates of four commonly used bracket bonding techniques (SEP, CM-AEP, SCR, and RM-GIC) and to analyse the time dependence of these failures.

A previous meta-analysis by Fleming *et al.* in 2012 indicated that the SEP method is inferior to the CM-AEP bonding technique (14), while by Namdari *et al.* (16) in 2021 indicated no difference in bracket debonding between these techniques. But none of these studies investigated the time dependence of bracket failure, an important factor when we consider the considerable length of orthodontic treatments. In the present work, by including more studies and creating groups based on follow-up periods we were able to show that SEP and conventional method (CM-AEP) did not demonstrate significant difference in failure rate at either 6, 12, or 18 months after bonding. However, the sensitivity analysis indicated that exclusion of the study of Aljubouri *et al.* (9). significantly affected the outcome of our analysis at 12 months. In the case of exclusion the difference between the two methods became significant, favouring CM-AEP application. After in-depth analysis we decided to not to exclude this study from our analysis, since we could not reveal any substantial difference in methodology between this work (9) and the rest of the included investigations. On the other hand, the studies of Elekdag-Turk *et al.* (45), Ireland *et al.* (50), and Murfitt *et al.* (23) clearly favoured the acid-etch technique and more risk in the SEP group. The trial of House *et al.* was not included into either meta-analysis due to the high level of failure risk (72.4%) reported, most probably because they used a different material (Ideal 1, GAC) compared with the others. In almost all included studies, similar primers (Transbond XT Primer, MIP, or Transbond Plus SEP, 3M) and composite paste materials (Transbond XT or APC bracket, 3M) from the main global manufacturer (3M) were used. The single exception was Pandis *et al.*, who preferred other products (Ortho Solo Primer and Enlight) from another manufacturer (Ormco) and the conventional method.

Taken together, our results suggest that the SEP bonding technique could be regarded as first choice and favourable during orthodontic intervention, since this method consists of fewer steps than CM-AEP thus requires less time (16), and less vulnerable to errors originating from operator usage (54). But the heterogeneity of outcomes indicate that more well designed, high-quality RCTs are needed to obtain strong evidence about the relative failure risk of bonding between the SEP the CM-AEP methods.

Both SCRs and simple or resin-modified glass ionomer materials present disadvantages compared with the etching techniques. These include cumbersome preparation of cement, possible displacement of bracket from its initial position during polymerization of SCR or RM-GIC, long polymerization time, and increases in operator dependency (55). Manual mixing of materials also increases the chance of errors (e.g. introduction of voids, alteration in proportions) (56). These and the presence of better alternatives are the main reasons why both techniques were pushed into the background in the field of orthodontics. Despite of all these, these chemically cured materials are in routine use during multibond fixed appliance therapy, when no alternative techniques can be applied. As a consequence, according to a survey, SCR, and RM-GIC materials are still used routinely in 23.6 and 3.4%, respectively, in orthodontics in the UK (57). In the case of band cementation, the use of GIC-based materials is still the best available technique, as etching is not an applicable option in such cases (57). During surgical exposure of impacted or unerupted teeth, the direct bond of attachment (standard bracket, button, hook) often fails following the use of adhesive techniques (58). When the control of moisture is limited or not possible, RM-GIC could be a good technique, as it is less sensitive in a wet environment and has clinically acceptable bond strength, which increases 24 hours after application (5,57). In such cases, the direct bonding of brackets with this material is not contraindicated, reportedly yielding acceptable or comparable results to composite adhesives (59–62).

In our meta-analysis, the risk of bond failure using SCR materials in comparison with GIC or resin-modified GIC was significantly lower at both 12 and 18 months after bonding. The difference in failing brackets was 6.0 and 20.8% after 12 and 18 months, respectively, representing dramatic disadvantages of RM-GIC over SCR, especially during at least one and a half year long applications. Our results are in accordance with the outcome of most individual investigations. Additionally, in vitro studies on bond strength also yielded lower values for glass ionomers than for adhesive technologies (7,63,64). Our results are substantially different from the outcome of the earlier meta-analysis by Khan *et al.*, showing that there was no statistically significant risk difference of bracket failure between brackets bonded with RM-GIC and SCR techniques (17). The reason for the divergent results are not clear. On the other hand, similar to us, Mickenautsch *et al.* (15) found no difference in the failure rate between the two treatment groups after 12 months and found in favour of composite resin adhesive after more than 14 months. However, in both studies the strength of evidence was much lower compared with us, since we were able to include considerable more studies. Also, the time-dependent breakdown in forming groups seems to be very important since bracket failure increase by time, as shown by many studies including our present investigation.

### Strengths of the study

An important strength of this meta-analysis is that it revealed no significant difference between SEP and CM-AEP up to 18 months after application. In addition, evidence was provided the superiority of SCR over RM-GIC was evident. Indirectly we were also able to compare CM-AEP, SEP, SCR, and RM-GIC techniques. The results clearly showed that glass ionomers had much worse failure rates compared with acid-etching methods thereby demonstrating an important message for clinicians for selecting their orthodontic bond technique. Additionally, subgroup analyses showed that in our investigation the outcomes did not depend on relatively small differences in study design.

### Limitations of the study

A major limitation of the present work is that the included clinical trials, with no exceptions, showed variable levels of risk of bias. The measurable variables of bonding processes presented in the summary table, such as light-curing time, etching time, and type of etch in the case of GIC and RM-GIC, also show some grade of differences among the involved studies. Also, demographic data are not uniformly presented and were incomplete in some cases. Other factors, such as the experience of the clinician, and the eating, chewing and tooth brushing habits of the patients—which also contribute to bracket failure—are not measurable and they were obviously variable in the included studies. The wide range of publication dates and the large scale of applied dental materials also cause a considerable limitation of our work. Additionally, due to the small number of available studies, we were unable to directly conduct a meta-analysis on the comparison of both CM-AEP, SEP, SCR, and RM-GIC techniques. Subgroup analyses could not be done in all study cohorts due to the low number of studies in certain experimental designs. Although multiple studies stated the importance of cluster analysis using available methodological and statistical approaches (65), no intra-cluster coefficients were reported in the included studies.

A possible problem affecting the outcome is the difference between the clustering effects of the S-M and the P-G bracket allocation methods. The S-M design may eliminate some of the unknown confounding factors, but it may introduce carry-over effects between different sides that are being evaluated (66). According to the Cochrane Handbook, the effects of clustering could be approximated by modifying the sample size and the number of subjects with effects based on the size of the cluster and the intra-cluster coefficient (67). It is desirable to pool separately the outcome measurements of S-M and P-G studies. But unfortunately, in our meta-analysis the number of studies with P-G bracket allocation was so low that only one subgroup analysis could be performed regarding SEP versus CM-AEP, which was at 12 months.

## Conclusions

Based on the results, the, null hypothesis regarding to the CM-AEP and SEP techniques has been accepted. Within the limitations of this meta-analysis, the results suggest no significant difference in bracket failure at 6, 12, and more than 18 months using the CM-AEP and SEP methods. In the case of SCR—RM-GIC techniques, the null hypothesis was rejected. The RM-GIC techniques performed worse at both the 12- and 18-month follow-up periods compared with self-cure composites. Based on our observations on bracket failure, in clinical applications lasting longer than 12 months, the CM-AEP and SEP techniques yielded three to six times better outcomes than the GIC and RM-GIC applications. Therefore, whenever it is possible, the first two techniques should be used over the latter two in clinical settings.

## Data availability

The data that support the findings of this study are available from the corresponding authors, upon reasonable request.

## Supplementary Material

cjac050_suppl_Supplementary_Figure_S1

cjac050_suppl_Supplementary_Figure_S2

cjac050_suppl_Supplementary_Figure_S3

cjac050_suppl_Supplementary_Figure_S4

cjac050_suppl_Supplementary_Figure_S5

cjac050_suppl_Supplementary_Figure_S6

cjac050_suppl_Supplementary_Figure_S7

cjac050_suppl_Supplementary_Figure_S8

cjac050_suppl_Supplementary_Table_S1

cjac050_suppl_Supplementary_Table_S2

cjac050_suppl_Supplementary_Table_S3

cjac050_suppl_Supplementary_Table_S4

cjac050_suppl_Supplementary_Table_S5

cjac050_suppl_Supplementary_Table_S6

cjac050_suppl_Supplementary_Table_S7

cjac050_suppl_Supplementary_Data

## Supplementary material

Supplementary Text 1. Qualitative analysis.

Supplementary Table 1. PRISMA checklist for abstract.

Supplementary Table 2. PRISMA checklist for main text.

Supplementary Table 3. Detailed search strategy.

Supplementary Table 4. Reasons of exclusion of records at full-text assessment.

Supplementary Table 5. Risk-of-bias assessment of RCTs.

Supplementary Table 6. Risk-of-bias assessment of non RCTs.

Supplementary Table 7. GRADE analysis.

Supplementary Figure 1. Sensitivity analysis of bracket failures between SEP and CM-AEP after 6 months. Omitting any of the studies did not significantly alter the overall estimate.

Supplementary Figure 2. Sensitivity analysis of bracket failures between SEP and CM-AEP after 12 months. Omitting the study, Aljubouri *et al.* (9) significantly changes the overall estimate [RR: 1.49 95% CI (1.10–2.03)].

Supplementary Figure 3. Sensitivity analysis of bracket failures between SEP and CM-AEP after more than 18 months. Omitting any of the records, did not significantly change the overall estimate.

Supplementary Figure 4. Sensitivity analysis of bracket failures between SCR and RM-GIC after 12 months. Leaving out any of the studies did not significantly change the overall estimate.

Supplementary Figure 5. Sensitivity analysis of bracket failures between SCR and RM-GIC after 18 months. Omitting any of the studies did not significantly alter the overall estimate.

Supplementary Figure 6. Funnel plot of SEP and CM-AEP after 6 months. Symmetrical funnel plot, with no signs of publication bias, Egger’s test (*P* = 0.890) indicates no small-study effect.

Supplementary Figure 7. Funnel plot of SEP and CM-AEP after 12 months. Missing dots (studies) indicates more studies are needed with increased sample size and statistical power, Egger’s test (*P* = 0.472) indicates no small-study effect.

Supplementary Figure 8. Funnel plot of SCR and RM-GIC after 12 months. Slight asymmetry of the plot indicates presence of statistical heterogeneity, however Egger’s test (*P* = 0.129) indicates no small-study effect.

## References

[1] Gange, P. (2015) The evolution of bonding in orthodontics. American Journal of Orthodontics and Dentofacial Orthopedics, 147, S56–S63. 25836345 10.1016/j.ajodo.2015.01.011

[2] Skidmore, K.J., Brook, K.J., Thomson, W.M. and Harding, W.J. (2006) Factors influencing treatment time in orthodontic patients. American Journal of Orthodontics and Dentofacial Orthopedics, 129, 230–238. 16473715 10.1016/j.ajodo.2005.10.003

[3] Brown, K. (2009) The impact of bonding material on bracket failure rate. Vital, 6, 28–30.

[4] Chinvipas, N. and Hasegawa, Y. (2014) Repeated bonding of fixed retainer increases the risk of enamel fracture. Odontology, 102, 89–97. 23239387 10.1007/s10266-012-0095-9

[5] Chaudhari, P.K., Goyal, L., Rana, S.S., Dhingra, K. and Kshetrimayum, N. (2018). Nanocomposites and nanoionomers for orthodontic bracket bonding. In Asiri A.M., Mohammad A. (eds.), Applications of Nanocomposite Materials in Dentistry. Woodhead Publishing Series in Biomaterials, Elsevier, pp. 171–180.

[6] Gibas-Stanek, M. and Pihut, M. (2021) Safe debonding of fixed appliances: a comparison of traditional techniques and LODI devices on different bracket types in terms of enamel cracks, site of bond failure and bracket reusability. International Journal of Environmental Research and Public Health, 18, 10267. 34639565 10.3390/ijerph181910267 PMC8508270

[7] Bishara, S.E., Vonwald, L. and Olsen, M.E. (1999) Effect of time on the shear bond strength of glass ionomer and composite orthodontic adhesives. American Journal of Orthodontics and Dentofacial Orthopedics, 116, 616–620. 10587594 10.1016/s0889-5406(99)70195-2

[8] e Cal-Neto, J.P., Quintão, C.A., de Oliveira Almeida, M.A. and Miguel, J.A.M. (2009) Bond failure rates with a self-etching primer: a randomized controlled trial. American Journal of Orthodontics and Dentofacial Orthopedics, 135, 782–786. 19524839 10.1016/j.ajodo.2008.11.022

[9] Aljubouri, Y.D., Millett, D.T. and Gilmour, W.H. (2004) Six and 12 months’ evaluation of a self-etching primer versus two-stage etch and prime for orthodontic bonding: a randomized clinical trial. European Journal of Orthodontics, 26, 565–571. 15650064 10.1093/ejo/26.6.565

[10] Banks, P. and Thiruvenkatachari, B. (2007) Long-term clinical evaluation of bracket failure with a self-etching primer: a randomized controlled trial. Journal of Orthodontics, 34, 243–251. 18042825 10.1179/146531207225022293

[11] Elekdag-Turk, S., Isci, D., Turk, T. and Cakmak, F. (2008) Six-month bracket failure rate evaluation of a self-etching primer. European Journal of Orthodontics, 30, 211–216. 18216373 10.1093/ejo/cjm119

[12] Wilson, A.D. and Kent, B.E. (1972) A new translucent cement for dentistry. The glass ionomer cement. British Dental Journal, 132, 133–135. 4501690 10.1038/sj.bdj.4802810

[13] Millett, D.T. and Mccabe, J.F. (1996) Orthodontic bonding with glass ionomer cement—a review. European Journal of Orthodontics, 18, 385–399. 8921660 10.1093/ejo/18.4.385

[14] Fleming, P.S., Johal, A. and Pandis, N. (2012) Self-etch primers and conventional acid-etch technique for orthodontic bonding: a systematic review and meta-analysis. American Journal of Orthodontics and Dentofacial Orthopedics, 142, 83–94. 22748994 10.1016/j.ajodo.2012.02.023

[15] Mickenautsch, S., Yengopal, V. and Banerjee, A. (2012) Retention of orthodontic brackets bonded with resin-modified GIC versus composite resin adhesives—a quantitative systematic review of clinical trials. Clinical Oral Investigations, 16, 1–14. 22006128 10.1007/s00784-011-0626-8

[16] Namdari, M., Amdjadi, P., Bayat, A., Seifi, M. and Alzwghaibi, A. (2021) Comparison of the failure rate, bonding time and ARI score of two orthodontic bonding systems: Self-Etch Primer and Conventional Etching Primer: a systematic review and meta-analysis. International Orthodontics, 19, 566–579. 34629307 10.1016/j.ortho.2021.09.001

[17] Khan, A.R., Fida, M. and Gul, M. (2020) Decalcification and bond failure rate in resin modified glass ionomer cement versus conventional composite for orthodontic bonding: a systematic review & meta-analysis. International Orthodontics, 18, 32–40. 31882396 10.1016/j.ortho.2019.10.003

[18] Page, M.J., et al. (2021) The PRISMA 2020 statement: an updated guideline for reporting systematic reviews. BMJ, 372, n71. 33782057 10.1136/bmj.n71 PMC8005924

[19] Sterne, J.A.C., et al. (2019) RoB 2: a revised tool for assessing risk of bias in randomised trials. BMJ, 366, 1–8. 10.1136/bmj.l4898 31462531

[20] Sterne, J., Hernánd, M., Reeves, B., Savović, J., Berkman, N. and Viswanathan, M. (2016) ROBINS-I: a tool for assessing risk of bias in non-randomised studies of interventions. BMJ, 355. 10.1136/bmj.i4919 PMC5062054 27733354

[21] Puhan, M.A., et al. (2014) A GRADE Working Group approach for rating the quality of treatment effect estimates from network meta-analysis. BMJ, 349, g5630. 25252733 10.1136/bmj.g5630

[22] Manning, N., Chadwick, S.M., Plunkett, D. and Macfarlane, T.V. (2006) A randomized clinical trial comparing ‘one-step’ and ‘two-step’ orthodontic bonding systems. Journal of Orthodontics, 33, 276–283. 17142334 10.1179/146531205225021825

[23] Murfitt, P.G., Quick, A.N., Swain, M.V. and Herbison, G.P. (2006) A randomised clinical trial to investigate bond failure rates using a self-etching primer. European Journal of Orthodontics, 28, 444–449. 16763088 10.1093/ejo/cjl007

[24] Dandachli, M.G. (2015) Bond failure rate of MBT brackets bonded with either self-etching primer or resin modified glass ionomer vs conventional method—an in vivo study. Dental and Medical Problems, 52, 440–446.

[25] Atik, E., Gorucu-Coskuner, H. and Taner, T. (2018) Clinical performance of precoated brackets and self-etch bonding technique: a prospective comparative study. Clinical Oral Investigations, 23, 2813–2821. 30506226 10.1007/s00784-018-2746-x

[26] Pandis, N., Polychronopoulou, A. and Eliades, T. (2006) Failure rate of self-ligating and edgewise brackets bonded with conventional acid etching and a self-etching primer: a prospective in vivo study. The Angle Orthodontist, 76, 119–122. 16448280 10.1043/0003-3219(2006)076[0119:FROSAE]2.0.CO;2

[27] Dominguez, G.C., Tortamano, A., de Moura Lopes, L.V., Chibebe Catharino, P.C. and Morea, C. (2013) A comparative clinical study of the failure rate of orthodontic brackets bonded with two adhesive systems: Conventional and Self-Etching Primer (SEP). Dental Press Journal of Orthodontics, 18, 55–60. 10.1590/s2176-94512013000200014 23916432

[28] Noor Sam, A. and Asma, A.A. (2012) Effectiveness of self-etching primer versus conventional etch and bond technique in fixed orthodontic treatment. Sains Malaysiana, 41, 1051–1056.

[29] Ozer, M., Bayram, M., Dincyurek, C. and Tokalak, F. (2014) Clinical bond failure rates of adhesive precoated self-ligating brackets using a self-etching primer. The Angle Orthodontist, 84, 155–160. 23819593 10.2319/022013-149.1 PMC8683055

[30] Reis, A., dos Santos, J.E., Longuercio, A.D. and de Oliveira Bauer, J.R. (2008) Eighteen-month bracket survival rate: conventional versus self-etch adhesive. European Journal of Orthodontics, 30, 94–99. 17989121 10.1093/ejo/cjm089

[31] Gaworski, M., Weinstein, M. and Borislow, A.J. (1999) Decalcification and bond failure: a comparison of a glass ionomer and a composite resin bonding system in vivo. American Journal of Orthodontics and Dentofacial Orthopedics, 116, 518–521. 10547510 10.1016/s0889-5406(99)70182-4

[32] Cacciafesta, V., Bosch, C. and Melsen, B. (1999) Clinical comparison between a resin-reinforced self-cured glass ionomer cement and a composite resin for direct bonding of orthodontic brackets. Part 2: bonding on dry enamel and on enamel soaked with saliva. Clinical Orthodontics and Research, 2, 186–193. 10806942 10.1111/ocr.1999.2.4.186

[33] Fricker, J.P. (1992) A 12-month clinical evaluation of a glass polyalkenoate cement for the direct bonding of orthodontic brackets. American Journal of Orthodontics and Dentofacial Orthopedics, 101, 381–384. 1558068 10.1016/S0889-5406(05)80332-4

[34] Fricker, J.P. (1994) A 12-month clinical evaluation of a light-activated glass polyalkenoate (ionomer) cement for the direct bonding of orthodontic brackets. American Journal of Orthodontics and Dentofacial Orthopedics, 105, 502–505. 8166101 10.1016/S0889-5406(94)70012-5

[35] Fricker, J.P. (1998) A new self-curing resin-modified glass-ionomer cement for the direct bonding of orthodontic brackets in vivo. American Journal of Orthodontics and Dentofacial Orthopedics, 113, 384–386. 9563352

[36] Hegarty, D.J. and Macfarlane, T.V. (2002) In vivo bracket retention comparison of a resin-modified glass ionomer cement and a resin-based bracket adhesive system after a year. American Journal of Orthodontics and Dentofacial Orthopedics, 121, 496–501. 12045767 10.1067/mod.2002.122367

[37] Miguel, J.A.M., Almeida, M.A. and Chevitarese, O. (1995) Clinical comparison between a glass ionomer cement and a composite for direct bonding of orthodontic brackets. American Journal of Orthodontics and Dentofacial Orthopedics, 107, 484–487. 7733057 10.1016/s0889-5406(95)70115-x

[38] Millett, D.T., Nunn, J.H., Welbury, R.R. and Gordon, P.H. (1999) Decalcification in relation to brackets bonded with glass ionomer cement or a resin adhesive. The Angle Orthodontist, 69, 65–70. 10022187 10.1043/0003-3219(1999)069<0065:DIRTBB>2.3.CO;2

[39] Fowler, P.V. (1998) A twelve-month clinical trial comparing the bracket failure rates of light-cured resin-modified glass-ionomer adhesive and acid-etch chemical-cured composite. Australian Orthodontic Journal, 15, 186–190. 10204428

[40] Miller, J.R., Arbuckle, G. and Phillips, R. (1996) A three-year clinical trial using a glass ionomer cement for the bonding of orthodontic brackets. The Angle Orthodontist, 66, 309–312. 8863967 10.1043/0003-3219(1996)066<0309:ATYCTU>2.3.CO;2

[41] Ousehal, L., El Aouame, A., Rachdy, Z. and Benkiran, G. (2016) Comparison of the efficacy of a conventional primer and a self-etching primer. International Orthodontics, 14, 195–205. 10.1016/j.ortho.2016.03.005 27080598

[42] Norevall, L.I., Marcusson, A. and Persson, M. (1996) A clinical evaluation of a glass ionomer cement as an orthodontic bonding adhesive compared with an acrylic resin. European Journal of Orthodontics, 18, 373–384. 8921659 10.1093/ejo/18.4.373

[43] Oliveira, S.R., Rosenbach, G., Brunhard, IHVP, Almeida, M.A. and Chevitarese, O. (2004) A clinical study of glass ionomer cement. European Journal of Orthodontics, 26, 185–189. 15130042 10.1093/ejo/26.2.185

[44] Ireland, A.J. and Sherriff, M. (2002) The effect of pumicing on the in vivo use of a resin modified glass poly(alkenoate) cement and a conventional no-mix composite for bonding orthodontic brackets. Journal of Orthodontics, 29, 217–220. 12218200 10.1093/ortho/29.3.217

[45] Elekdag-Turk, S., Cakmak, F., Isci, D. and Turk, T. (2008) 12-Month self-ligating bracket failure rate with a self-etching primer. The Angle Orthodontist, 78, 1095–1100. 18947290 10.2319/112507-552.1

[46] dos Santos, J.E., Quioca, J., Loguercio, A.D. and Reis, A. (2006) Six-month bracket survival with a self-etch adhesive. The Angle Orthodontist, 76, 863–868. 17029523 10.1043/0003-3219(2006)076[0863:SBSWAS]2.0.CO;2

[47] Romano, F.L., Valério, R.A., Gomes-Silva, J.M., Ferreira, J.T.L., Faria, G. and Borsatto, M.C. (2012) Clinical evaluation of the failure rate of metallic brackets bonded with orthodontic composites. Brazilian Dental Journal, 23, 399–402. 23207856 10.1590/s0103-64402012000400015

[48] Cal-Neto, J.P.E. and Miguel, J.A.M. (2005) An in vivo evaluation of bond failure rates with hydrophilic and self-etching primer systems. Journal of Clinical Orthodontics, 39, 701–702. 16456306

[49] Asgari, S., Salas, A., English, J. and Powers, J. (2002) Clinical evaluation of bond failure rates with a new self-etching primer. Journal of Clinical Orthodontics, 36, 687–689. 12572254

[50] Ireland, A.J., Knight, H. and Sherriff, M. (2003) An in vivo investigation into bond failure rates with a new self-etching primer system. American Journal of Orthodontics and Dentofacial Orthopedics, 124, 323–326. 12970667 10.1016/s0889-5406(03)00403-7

[51] Fricker, J.P. (1992) A 12-month clinical evaluation of a glass polyalkenoate cement for the direct bonding of orthodontic brackets. American Journal of Orthodontics and Dentofacial Orthopedics, 101, 381–384. 1558068 10.1016/S0889-5406(05)80332-4

[52] Romano, F.L., Correr, A.B., Correr-Sobrinho, L., Magnani, M.B.B.A. and Ruellas, A.C.O. (2012) Clinical evaluation of the failure rates of metallic brackets. Journal of Applied Oral Science, 20, 228–234. 22666842 10.1590/S1678-77572012000200018 PMC3894768

[53] Ok, U., Aksakalli, S., Eren, E. and Kechagia, N. (2021) Single-component orthodontic adhesives: comparison of the clinical and in vitro performance. Clinical Oral Investigations, 25, 3987–3999. 33404765 10.1007/s00784-020-03729-z

[54] Zope, A., et al. (2016) Comparison of self-etch primers with conventional acid etching system on orthodontic brackets. Journal of Clinical and Diagnostic Research, 10, ZC19–ZC22. 10.7860/JCDR/2016/18842.9031 PMC5296570 28208997

[55] Galindo, H.R.A., Sadowsky, P.L., Vlachos, C., Jacobson, A. and Wallace, D. (1998) An in vivo comparison between a visible light-cured bonding system and a chemically cured bonding system. American Journal of Orthodontics and Dentofacial Orthopedics, 113, 271–275. 9517717 10.1016/s0889-5406(98)70296-3

[56] Ash, S. and Hay, N. (1996) Adhesive pre-coated brackets, a comparative clinical study. British Journal of Orthodontics, 23, 325–329. 8985569 10.1179/bjo.23.4.325

[57] Banks, P., Elton, V., Jones, Y., Rice, P., Derwent, S. and Odondi, L. (2010) The use of fixed appliances in the UK: a survey of specialist orthodontists. Journal of Orthodontics, 37, 43–55. 20439926 10.1179/14653121042867

[58] Becker, A., Shpack, N. and Shteyer, A. (1996) Attachment bonding to impacted teeth at the time of surgical exposure. European Journal of Orthodontics, 18, 457–463. 8942094 10.1093/ejo/18.5.457

[59] Cacciafesta, V., Bosch, C. and Melsen, B. (1998) Clinical comparison between a resin-reinforced self-cured glass ionomer cement and a composite resin for direct bonding of orthodontic brackets. Part 1: wetting with water. Clinical Orthodontics and Research, 1, 29–36. 9918643 10.1111/ocr.1998.1.1.29

[60] Benson, P.E., et al. (2019) Resin-modified glass ionomer cement vs composite for orthodontic bonding: a multicenter, single-blind, randomized controlled trial. American Journal of Orthodontics and Dentofacial Orthopedics, 155, 10–18. 30591153 10.1016/j.ajodo.2018.09.005

[61] Choo, S.C., Ireland, A.J. and Sherriff, M. (2001) An in vivo investigation into the use of resin-modified glass poly(alkenoate) cements as orthodontic bonding agents. European Journal of Orthodontics, 23, 403–409. 11471267 10.1093/ejo/23.3.243

[62] Summers, A., Kao, E., Gillmore, J., Gunel, E. and Ngan, P. (2004) Comparison of bond strength between a conventional resin adhesive and a resin-modified glass ionomer adhesive: an in vitro and in vivo study. American Journal of Orthodontics and Dentofacial Orthopedics, 126, 200–206. 15316475 10.1016/j.ajodo.2003.06.013

[63] Bishara, S.E., Gordan, V.V., VonWald, L. and Jakobsen, J.R. (1999) Shear bond strength of composite, glass ionomer, and acidic primer adhesive systems. American Journal of Orthodontics and Dentofacial Orthopedics, 115, 24–28. 9878954 10.1016/s0889-5406(99)70312-4

[64] Movahhed, H.Z., Øgaard, B. and Syverud, M. (2005) An in vitro comparison of the shear bond strength of a resin-reinforced glass ionomer cement and a composite adhesive for bonding orthodontic brackets. European Journal of Orthodontics, 27, 477–483. 16043469 10.1093/ejo/cji051

[65] Oltean, H. and Gagnier, J. (2015) Use of clustering analysis in randomized controlled trials in orthopaedic surgery. BMC Medical Research Methodology, 15, 1–8. 25887529 10.1186/s12874-015-0006-1 PMC4359453

[66] Kerayechian, N., Bardideh, E. and Bayani, S. (2022) Comparison of self-etch primers with conventional acid-etch technique for bonding brackets in orthodontics: a systematic review and meta-analysis. European Journal of Orthodontics, 44, 385–395. 35022707 10.1093/ejo/cjab076

[67] Higgins, J.P.T., Thomas, J., Chandler, J., Cumpston, M., Li, T., Page, M.J. and Welch, V.A. (2019) Cochrane Handbook for Systematic Reviews of Interventions. John Wiley & Sons, Chichester, UK, 2nd edn. 10.1002/14651858.ED000142 PMC10284251 31643080

