# Supplementary figures and images for: Clinical bracket failure rates between different bonding techniques: a systematic review and meta-analysis
Source: Eur J Orthod. 2022 Oct 12;45(2):175–85. doi: 10.1093/ejo/cjac050 (PMC10065138; doi:10.1093/ejo/cjac050)

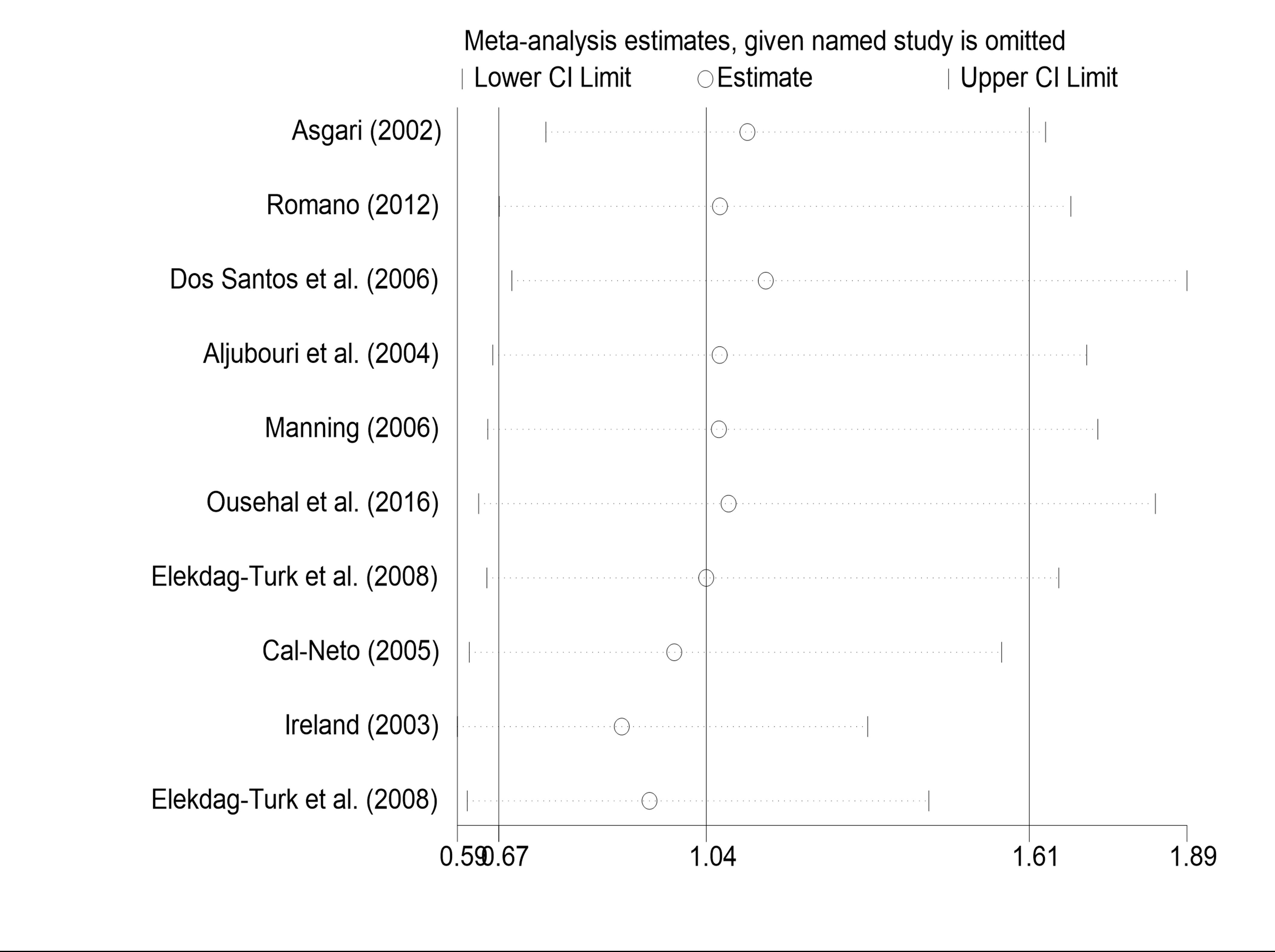

Supplement: cjac050_suppl_Supplementary_Figure_S1 [file cjac050_suppl_supplementary_figure_s1.jpeg]

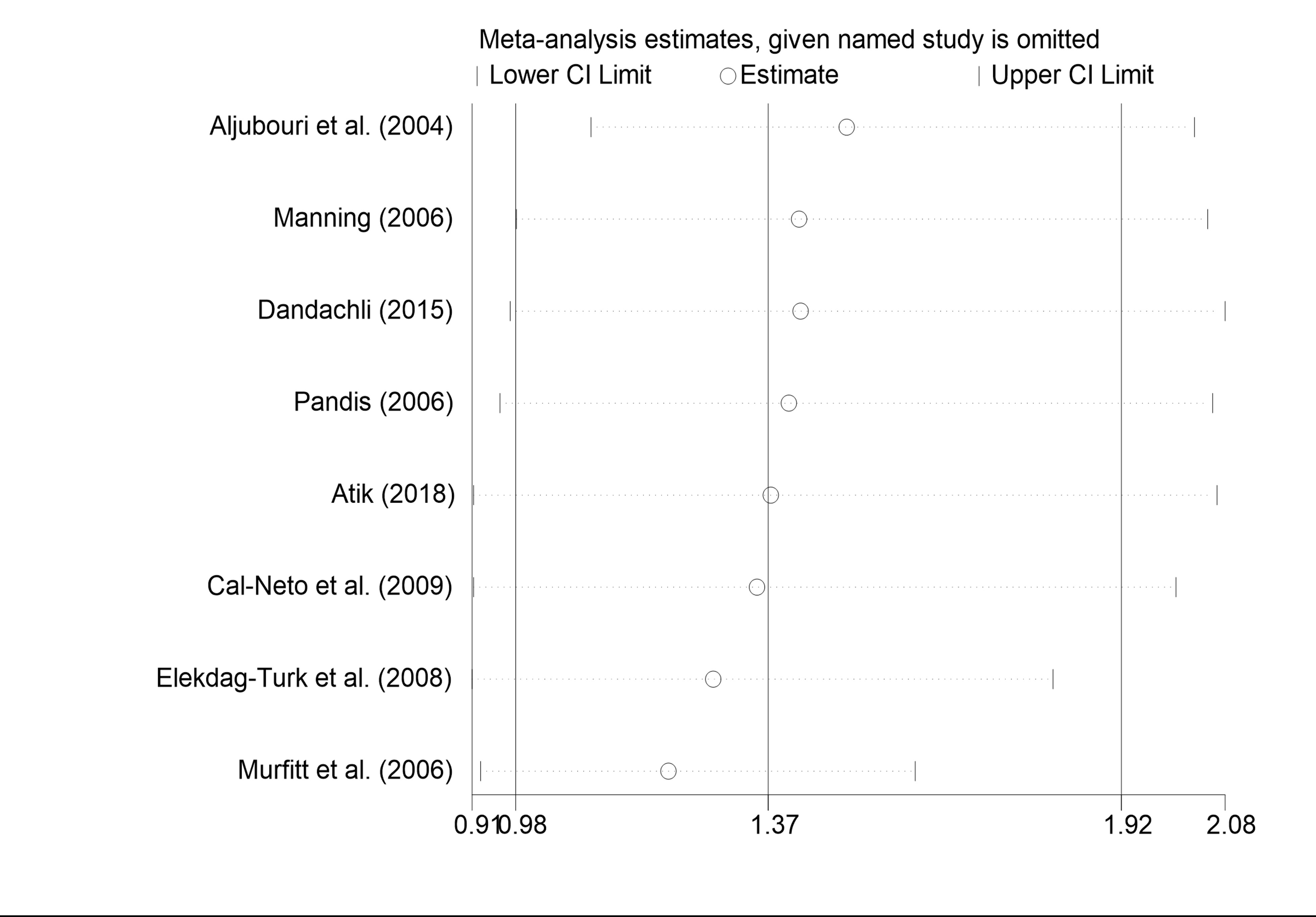

Supplement: cjac050_suppl_Supplementary_Figure_S2 [file cjac050_suppl_supplementary_figure_s2.jpeg]

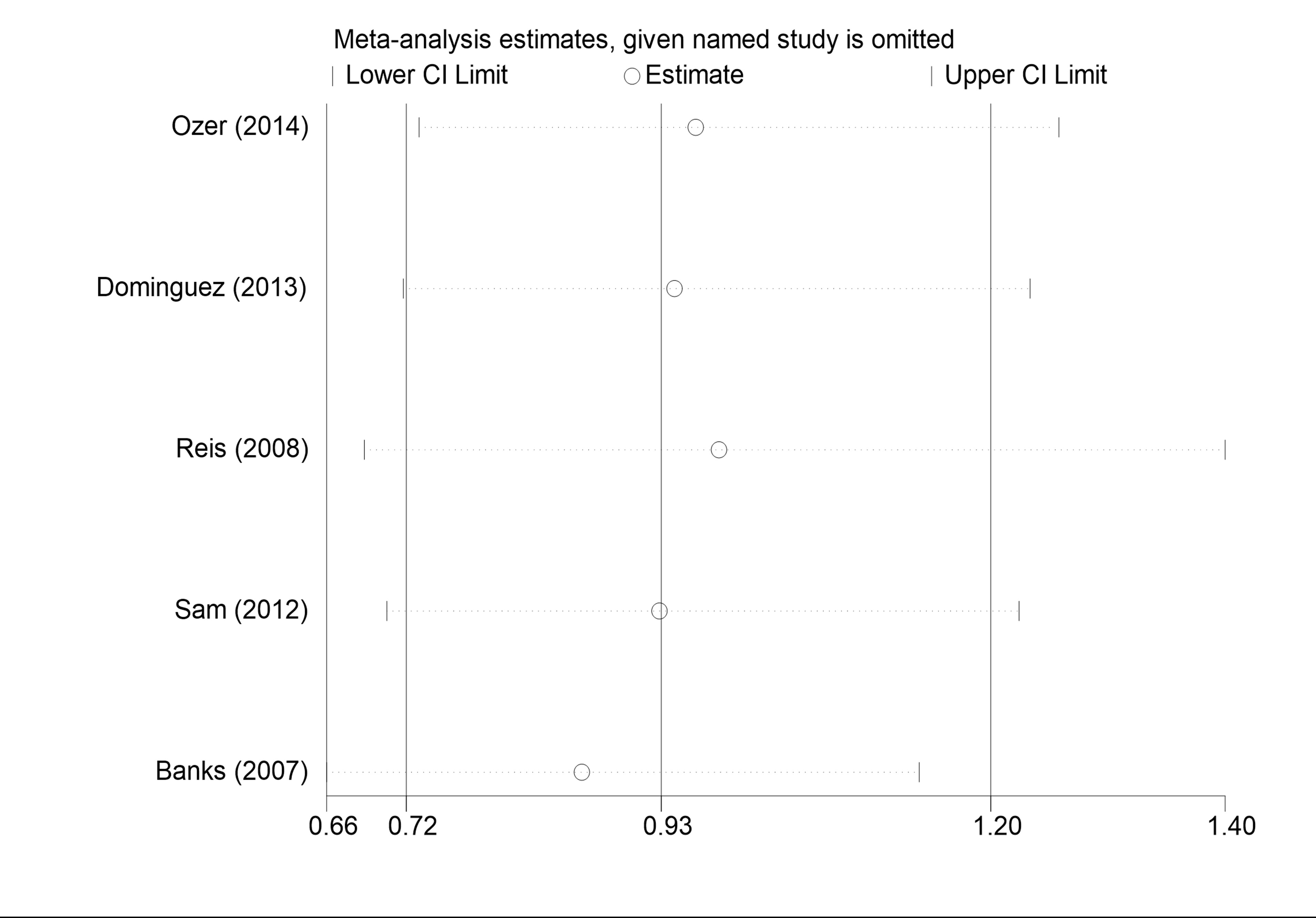

Supplement: cjac050_suppl_Supplementary_Figure_S3 [file cjac050_suppl_supplementary_figure_s3.jpeg]

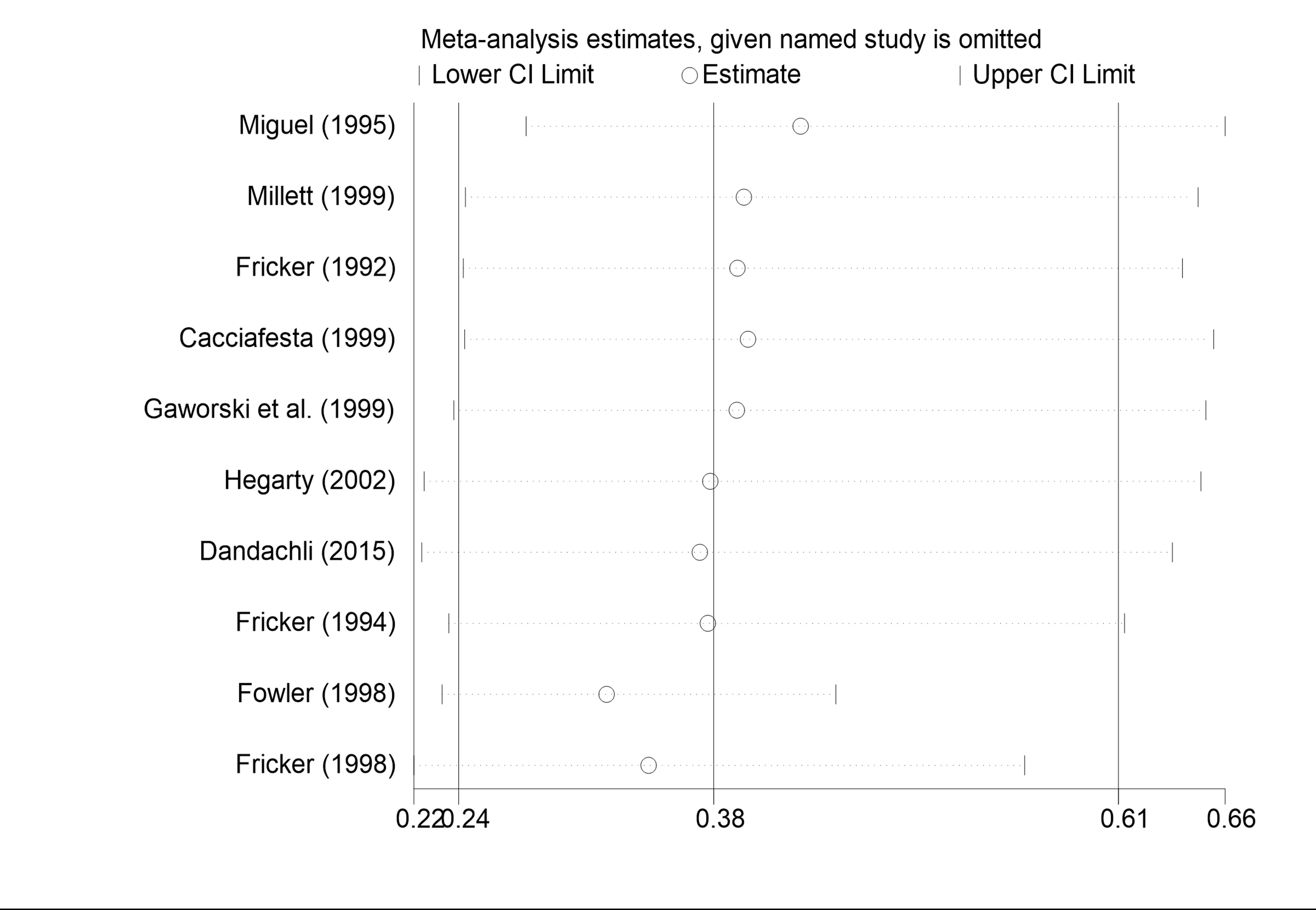

Supplement: cjac050_suppl_Supplementary_Figure_S4 [file cjac050_suppl_supplementary_figure_s4.jpeg]

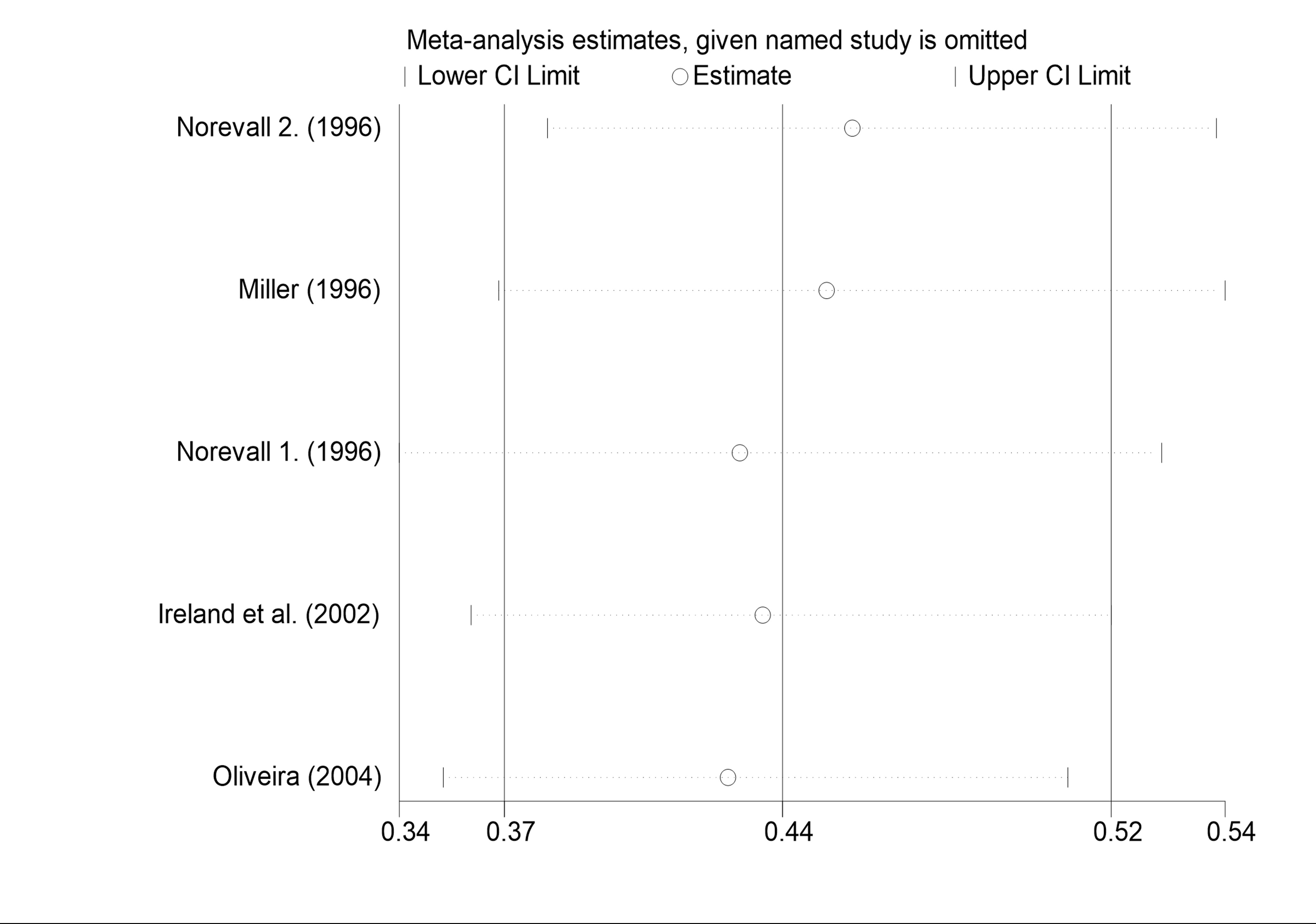

Supplement: cjac050_suppl_Supplementary_Figure_S5 [file cjac050_suppl_supplementary_figure_s5.jpeg]

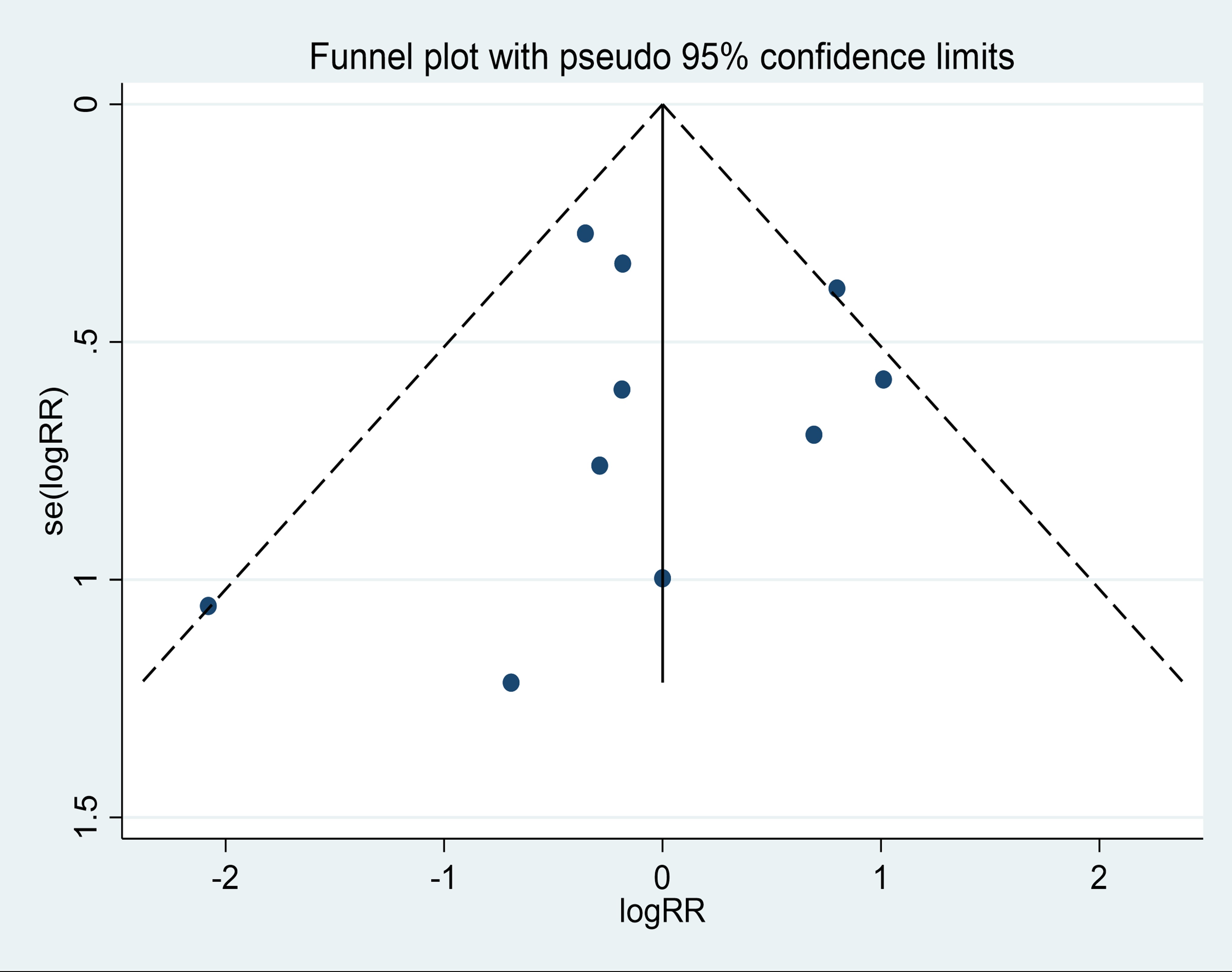

Supplement: cjac050_suppl_Supplementary_Figure_S6 [file cjac050_suppl_supplementary_figure_s6.jpeg]

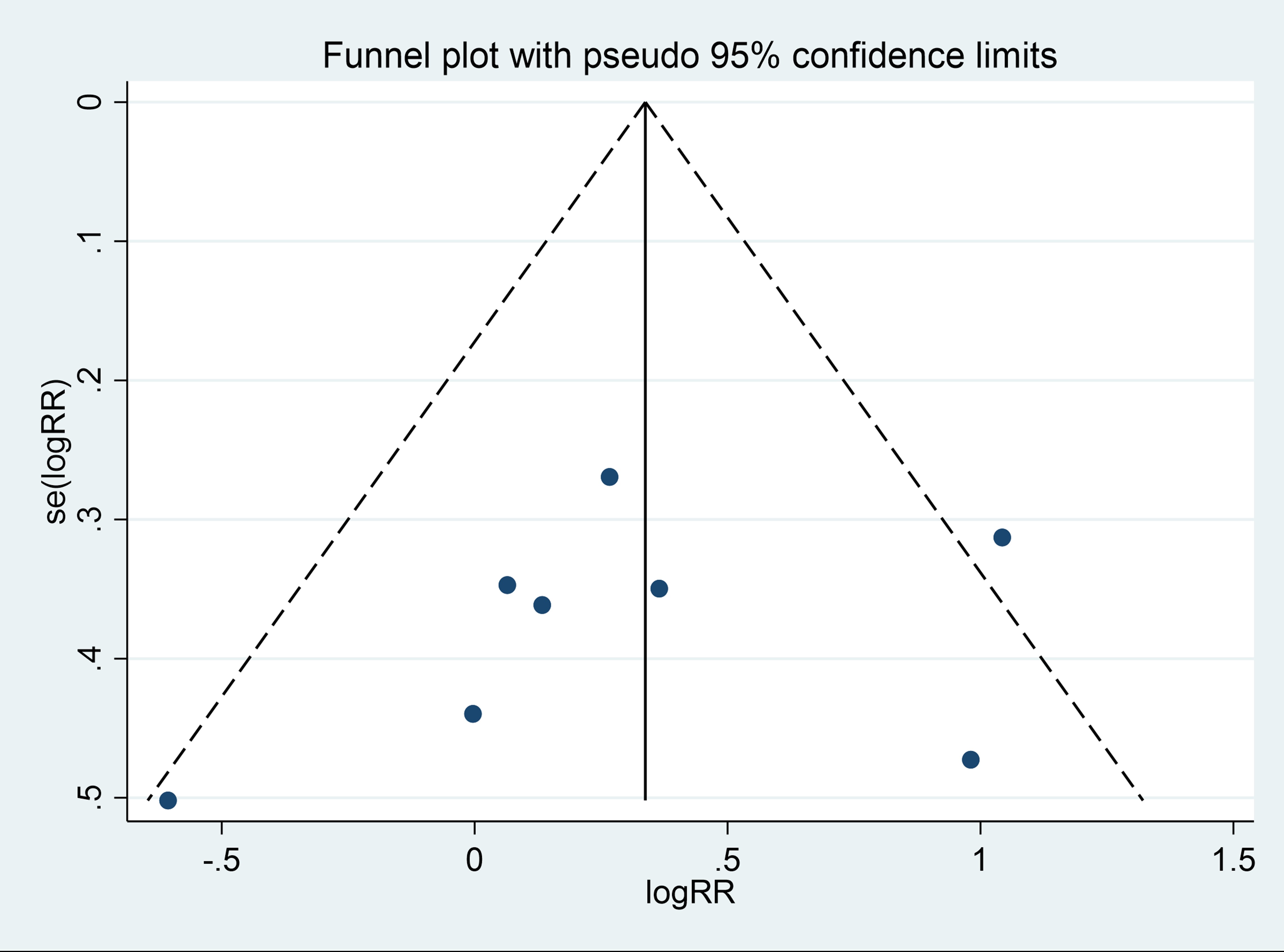

Supplement: cjac050_suppl_Supplementary_Figure_S7 [file cjac050_suppl_supplementary_figure_s7.jpeg]

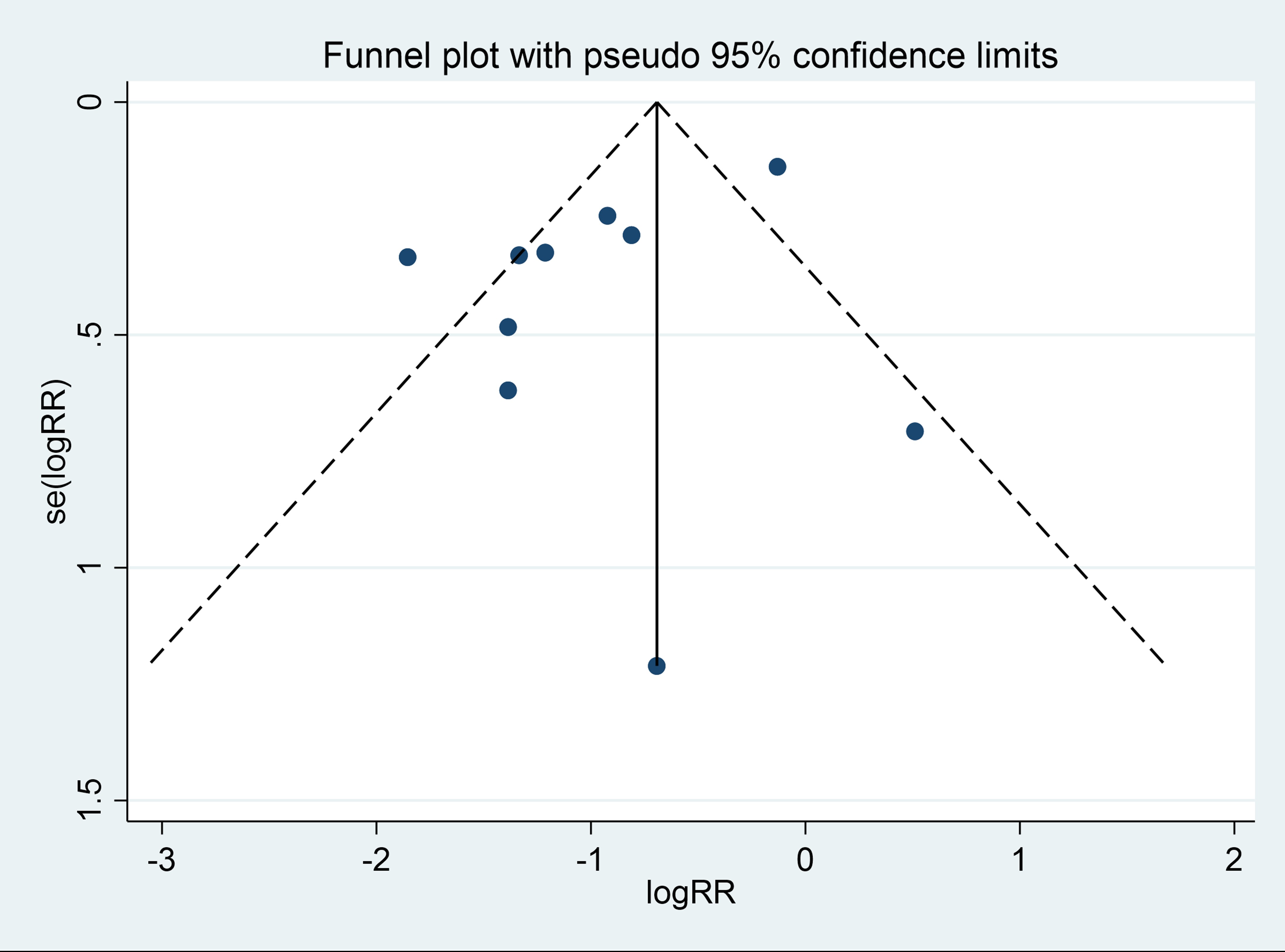

Supplement: cjac050_suppl_Supplementary_Figure_S8 [file cjac050_suppl_supplementary_figure_s8.jpeg]
