# Supplementary material for: Clinical bracket failure rates between different bonding techniques: a systematic review and meta-analysis
Source: Eur J Orthod. 2022 Oct 12;45(2):175–85. doi: 10.1093/ejo/cjac050 (PMC10065138; doi:10.1093/ejo/cjac050)
Supplement: cjac050_suppl_Supplementary_Table_S3 [file cjac050_suppl_supplementary_table_s3.docx]

| **MEDLINE (via PubMed)** | |
| --- | --- |
| Date of search | 22.10.2021 |
| Field of search | All fields |
| Filters and restrictions | no |
| Search key | ((glass ionomer) OR (dual cure) OR (self cure) OR (SEP OR self etch OR self etching) OR (acid etch OR two-step etch OR bonding agent OR primer)) AND (bracket OR braces OR brackets) |
| Number of records | 3 242 |
| **Embase** | |
| Date of search | 22.10.2021 |
| Field of search | All fields |
| Filters and restrictions | no |
| Search key | ((glass ionomer) OR (dual cure) OR (self cure) OR (SEP OR self etch OR self etching) OR (acid etch OR two-step etch OR bonding agent OR primer)) AND (bracket OR braces OR brackets). |
| Number of records | 1 305 |
| **Cochrane Central Register of Controlled Trials (CENTRAL)** | |
| Date of search | 22.10.2021 |
| Field of search | All fields |
| Filters and restrictions | no |
| Search key | ((glass ionomer) OR (dual cure) OR (self cure) OR (SEP OR self etch OR self etching) OR (acid etch OR two-step etch OR bonding agent OR primer)) AND (bracket OR braces OR brackets). |
| Number of records | 1 591 |
