# Supplementary material for: Clinical bracket failure rates between different bonding techniques: a systematic review and meta-analysis
Source: Eur J Orthod. 2022 Oct 12;45(2):175–85. doi: 10.1093/ejo/cjac050 (PMC10065138; doi:10.1093/ejo/cjac050)
Supplement: cjac050_suppl_Supplementary_Table_S4 [file cjac050_suppl_supplementary_table_s4.docx]

| **Study** | **Reason of exclusion** |
| --- | --- |
| Al Maaitah et al. 2019: Effect of fixed orthodontic appliances bonded  with different etching techniques on tooth color: A prospective clinical study | different examined outcome |
| Artun et al. 1984: Clinical trials with crystal growth conditioning as an alternative to acid-etch enamel pretreatment | inadequate design for inclusion |
| Artun 1997: A post-treatment evaluation of multibonded ceramic brackets in orthodontics | inadequate design for inclusion |
| Banks et al. 1994: Enamel sealants: a clinical evaluation of their value during fixed appliance therapy | inadequate design for inclusion |
| Banks et al. 1997: A clinical evaluation of the effectiveness of including fluoride into an orthodontic bonding adhesive | inadequate design for inclusion, different examined outcome |
| Cal-Neto et al. 2006: Effect of a Self-etching Primer on Shear Bond Strength of Adhesive Precoated Brackets In Vivo | inadequate design for inclusion, different examined outcome |
| Hussein et al., 2014: The Impact of Chlorhexidine Mouth Rinse on the Bond Strength of Polycarbonate Orthodontic Brackets | inadequate design for inclusion, different examined outcome |
| Gorelick 1977: Bonding metal brackets with a self-polymerizing sealant-composite: A 12-month assessment | inadequate design for inclusion |
| Ireland et al. 1997: The Effect of Timing of Archwire Placement  on in vivo Bond Failure | inadequate design for inclusion |
| Marcusson et al. 1997: White spot reduction when using glass ionomer cement for bonding in orthodontics: a longitudinal and comparative study | different examined outcome |
| Oeiras et al. 2016: Survival analysis of banding and  bonding molar tubes in adult patients over a 12-month period: a split-mouth randomized clinical trial | different examined outcome |
| Oz et al. 2019: Twenty-four-month clinical performance of different universal adhesives in etch-and-rinse, selective etching and self-etch application modes in NCCL – a randomized controlled clinical trial | different examined outcome |
| Retief et al. 1975: Clinical experience with the acid-etch technique in orthodontics | inadequate design for inclusion |
| Sharma et al. 2013: A comparative evaluation of the retention of metallic brackets bonded with resin‑modified glass ionomer cement under different enamel preparations: A pilot study | inadequate design for inclusion |
| Silverman et al. 1995: A new light-cured glass ionomer cement that bonds brackets to teeth without etching in the presence of saliva, | inadequate design for inclusion |
| Springate et al. 1991: An Evaluation of Zirconium Oxide Brackets: A  Preliminary Laboratory and Clinical Report | inadequate design for inclusion |
| Uysal et al. 2011: Effect of antibacterial monomer-containing adhesive on enamel demineralization around orthodontic brackets: An in-vivo study | different examined outcome |
| Vig et al. 2019: Particulate production during debonding  of fixed appliances: Laboratory investigation and randomized clinical  trial to assess the effect of using flash-free ceramic brackets | inadequate design for inclusion |
| Voss et al. 1993: In vivo bonding of orthodontic brackets with glass ionomer cement | inadequate design for inclusion |
| Zachrisson et al. 1978: Clinical comparison of direct versus indirect bonding with different bracket types and adhesives | inadequate design for inclusion |
| Bozelli et al. 2013: Comparative study on direct and indirect bracket bonding techniques regarding time length and bracket detachment | indirect bonding technique |
| Dubernard et al. 2013: Comparative study of enamel adhesion  between RelyX Unicem (3M), a self-adhesive bonding agent, and the  combination of MIP (3M), a hydrophilic adhesive, and Transbond Supreme Low Viscosity (3M), a traditional hydrophobic adhesive | indirect bonding technique |
| Mavreas et al. 2008: The effect of various adhesives, enamel etching,  and base treatment on the failure frequency of customized lingual brackets: a randomized clinical trial | inadequate design for inclusion, indirect bonding technique |
| Miles et al. 2010: Indirect bonding – do custom bases need a plastic  conditioner? A randomised clinical trial | indirect bonding technique |
| Miles et al. 2008: Does microetching enamel reduce bracket  failure when indirect bonding mandibular  posterior teeth? | indirect bonding technique |
| Miles et al. 2005: A Comparison of Two Indirect Bonding Adhesives | indirect bonding technique |
| Miles et al. 2003: A clinical comparison of two chemically-cured adhesives used for indirect bonding | indirect bonding technique |
| Miles et al. 2000: A comparison of retention rates of brackets with thermally-cured and light-cured custom bases in indirect bonding procedures | indirect bonding technique |
| Read et al. 1990: A clinical trial of an indirect bonding technique with a visible light-cured adhesive | indirect bonding technique |
| Thiyagarajah et al. 2006: Aclinical comparison of bracket bond  failures in association with direct and indirect bonding | indirect bonding technique |
| Vijayakumar et al. 2014: How and why of orthodontic bond failures:  An in vivo study | indirect bonding technique |
| Hobson et al. 2002: The relationship between acid-etch patterns  and bond survival in vivo | inadequate design for inclusion |
| Kinch et al. 1989: A clinical study of amount of adhesive remaining on enamel after debonding, comparing etch times of 15 and 60 seconds | inadequate design for inclusion |
| Linklater et al. 2003: Bond failure patterns in vivo | inadequate design for inclusion |
| Millett et al. 1994: A 5-year clinical review of bond failure with a no-mix adhesive (Right on®) | inadequate design for inclusion |
| Nazir et al.2011: Banding versus bonding of first permanent molars: a multi-centre randomized controlled trial | inadequate design for inclusion |
| Shamma et al. 1999: Comparison of bracket debonding force between two conventional resin adhesives and a resin-reinforced glass ionomer cement: an in vitro and in vivo studys | inadequate design for inclusion |
| Signorelli et al. 2006: Comparison of bond strength between orthodontic brackets bonded with halogen and plasma arc curing lights: An in-vitro and in-vivo study | inadequate design for inclusion |
| Verstrynge et al. 2004: Clinical comparison of an  adhesive precoated vs. an uncoated ceramic bracket system | inadequate design for inclusion |
| Wenderoth et al. 1999: Effectiveness of a fluoride-releasing sealant in reducing decalcification during orthodontic treatment | inadequate design for inclusion |
| Miller 2001: Laboratory and Clinical Evaluation of a Self-Etching Primer | inadequate design for inclusion |
| Jou et al. 1995: Bonding Ceramic Brackets with Light-Cured Glass Ionomer Cements | inadequate design for inclusion, different examined outcome |
| Atilla et al. 2019: A comparative assessment of orthodontic treatment outcomes using the quantitative light-induced fluorescence (QLF) method between direct bonding and indirect bonding techniques in adolescents: a single-centre, single-blind randomized controlled trial | different examined outcome, indirect bonding technique |
| Comert et al. 2019: Clinical effect of a fluoride-releasing and rechargeable primer in reducing white spot lesions during orthodontic treatment | inadequate design for inclusion |
| Ok et al. 2021: Single-component orthodontic adhesives: comparison of the clinical and in vitro performance | different examined outcome |
| Rossato et al. 2020: Do fluorescent agents alter the mechanical  strength of orthodontic adhesives? An in vitro and clinical study | different examined outcome |
| Vig et al. 2019: Particulate production during debonding of fixed appliances: Laboratory investigation and randomized clinical trial to assess the effect of using flash-free ceramic brackets | inadequate design for inclusion |
| Doss et al. 2020: Clinical Ef􀧎icacy of Fluoride-releasing Dental Adhesive on Restricting White Spot Lesion: An in vivo Study | inadequate design for inclusion |
| Bao-shan et al. 2018: An in vivo and in vitro study on the effect of tooth liquid polish sealant on the bond strength of orthodontic brackets | different examined outcome |
