# Supplementary material for: Clinical bracket failure rates between different bonding techniques: a systematic review and meta-analysis
Source: Eur J Orthod. 2022 Oct 12;45(2):175–85. doi: 10.1093/ejo/cjac050 (PMC10065138; doi:10.1093/ejo/cjac050)
Supplement: cjac050_suppl_Supplementary_Table_S5 [file cjac050_suppl_supplementary_table_s5.docx]

| \| Study ID \| Experimental \| Comparator \| Outcome \| Weight \| Randomization process \| Deviations from intended interventions \| Missing outcome data \| Measurement of the outcome \| Selection of the reported result \| Overall \|  \|  \|  \| \| --- \| --- \| --- \| --- \| --- \| --- \| --- \| --- \| --- \| --- \| --- \| --- \| --- \| --- \| \| Aljubouri, 2004 \| SEP \| CMAEP \| Bracket failure \| 1 \|  \|  \|  \|  \|  \|  \|  \|  \| Low risk \| \| Ousehal, 2016 \| SEP \| CM-AEP \| Bracket failure \| 1 \|  \|  \|  \|  \|  \|  \|  \|  \| Some concerns \| \| Elekdag-Turk, 2008 (1) \| SEP \| CM-AEP \| Bracket failure \| 1 \|  \|  \|  \|  \|  \|  \|  \|  \| High risk \| \| Elekdag-Turk, 2008 (2) \| SEP \| CM-AEP \| Bracket failure \| 1 \|  \|  \|  \|  \|  \|  \|  \|  \|  \| \| dos Santos, 2006 \| SEP \| CM-AEP \| Bracket failure \| 1 \|  \|  \|  \|  \|  \|  \|  \|  \|  \| \| Cal-Neto, 2005 \| SEP \| CM-AEP \| Bracket failure \| 1 \|  \|  \|  \|  \|  \|  \|  \|  \|  \| \| Asgari, 2002 \| SEP \| CM-AEP \| Bracket failure \| 1 \|  \|  \|  \|  \|  \|  \|  \|  \|  \| \| Ireland, 2003 \| SEP \| CM-AEP \| Bracket failure \| 1 \|  \|  \|  \|  \|  \|  \|  \|  \|  \| \| Manning, 2006 \| SEP \| CM-AEP \| Bracket failure \| 1 \|  \|  \|  \|  \|  \|  \|  \|  \|  \| \| Cal-Neto, 2009 \| SEP \| CMAEP \| Bracket failure \|  \|  \|  \|  \|  \|  \|  \|  \|  \|  \| \| Murfitt, 2006 \| SEP \| CMAEP \| Bracket failure \|  \|  \|  \|  \|  \|  \|  \|  \|  \|  \| \| Atik, 2018 \| SEP \| CM-AEP \| Bracket failure \|  \|  \|  \|  \|  \|  \|  \|  \|  \|  \| \| Dominguez, 2013 \| SEP \| CM-AEP \| Bracket failure \|  \|  \|  \|  \|  \|  \|  \|  \|  \|  \| \| Sam, 2012 \| SEP \| CM-AEP \| Bracket failure \|  \|  \|  \|  \|  \|  \|  \|  \|  \|  \| \| Reis, 2008 \| SEP \| CM-AEP \| Bracket failure \|  \|  \|  \|  \|  \|  \|  \|  \|  \|  \| \| Banks, 2007 \| SEP \| CM-AEP \| Bracket failure \|  \|  \|  \|  \|  \|  \|  \|  \|  \|  \| \| Cacciafesta, 1999 \| RMGIC \| SCR \| Bracket failure \|  \|  \|  \|  \|  \| \| Hegarty, 2002 \| RMGIC \| SCR \| Bracket failure \|  \|  \|  \|  \|  \|  \|  \|  \|  \|  \| \| Millett, 1999 \| RMGIC \| SCR \| Bracket failure \|  \|  \|  \|  \|  \| \| Miller, 1996 \| RMGIC \| SCR \| Bracket failure \|  \|  \|  \|  \|  \| \| Norevall, 1996 \| RMGIC \| SCR \| Bracket failure \|  \|  \|  \|  \|  \| \| Oliveira, 2004 \| RMGIC \| SCR \| Bracket failure \|  \|  \|  \|  \|  \| |  |  |  |  |  |  |  |  |  |  |  |  |  |
| --- | --- | --- | --- | --- | --- | --- | --- | --- | --- | --- | --- | --- | --- | --- | --- | --- | --- | --- | --- | --- | --- | --- | --- | --- | --- | --- | --- | --- | --- | --- | --- | --- | --- | --- | --- | --- | --- | --- | --- | --- | --- | --- | --- | --- | --- | --- | --- | --- | --- | --- | --- | --- | --- | --- | --- | --- | --- | --- | --- | --- | --- | --- | --- | --- | --- | --- | --- | --- | --- | --- | --- | --- | --- | --- | --- | --- | --- | --- | --- | --- | --- | --- | --- | --- | --- | --- | --- | --- | --- | --- | --- | --- | --- | --- | --- | --- | --- | --- | --- | --- | --- | --- | --- | --- | --- | --- | --- | --- | --- | --- | --- | --- | --- | --- | --- | --- | --- | --- | --- | --- | --- | --- | --- | --- | --- | --- | --- | --- | --- | --- | --- | --- | --- | --- | --- | --- | --- | --- | --- | --- | --- | --- | --- | --- | --- | --- | --- | --- | --- | --- | --- | --- | --- | --- | --- | --- | --- | --- | --- | --- | --- | --- | --- | --- | --- | --- | --- | --- | --- | --- | --- | --- | --- | --- | --- | --- | --- | --- | --- | --- | --- | --- | --- | --- | --- | --- | --- | --- | --- | --- | --- | --- | --- | --- | --- | --- | --- | --- | --- | --- | --- | --- | --- | --- | --- | --- | --- | --- | --- | --- | --- | --- | --- | --- | --- | --- | --- | --- | --- | --- | --- | --- | --- | --- | --- | --- | --- | --- | --- | --- | --- | --- | --- | --- | --- | --- | --- | --- | --- | --- | --- | --- | --- | --- | --- | --- | --- | --- | --- | --- | --- | --- | --- | --- | --- | --- | --- | --- | --- | --- | --- | --- | --- | --- | --- | --- | --- | --- | --- | --- | --- | --- | --- | --- | --- | --- | --- | --- | --- | --- | --- | --- | --- | --- | --- | --- | --- | --- | --- | --- | --- | --- | --- | --- | --- | --- | --- | --- | --- | --- | --- | --- | --- | --- | --- | --- | --- | --- | --- | --- |
|  |  |  |  |  |  |  |  |  |  |  |  |  |  |
|  |  |  |  |  |  |  |  |  |  |  |  |  |  |
|  |  |  |  |  |  |  |  |  |  |  |  |  |  |
