# Supplementary material for: Clinical bracket failure rates between different bonding techniques: a systematic review and meta-analysis
Source: Eur J Orthod. 2022 Oct 12;45(2):175–85. doi: 10.1093/ejo/cjac050 (PMC10065138; doi:10.1093/ejo/cjac050)
Supplement: cjac050_suppl_Supplementary_Table_S6 [file cjac050_suppl_supplementary_table_s6.docx]

| **Reference** | **Type of study** | **Bias due to confounding** | **Bias in selection of participants** | **Bias in classification of interventions** | **Bias due to deviations from intended interventions** | **Bias due to missing data** | **Bias in measurement of outcome** | **Bias in selection of reported result** |
| --- | --- | --- | --- | --- | --- | --- | --- | --- |
| Gaworski, 1999 | Prospective cohort | Low | Low | Low | Low | Low | Moderate | Low |
| Dandachli, 2015 | Prospective cohort | Moderate | Moderate | Low | Low | Low | Moderate | Low |
| Fricker, 1992 | Prospective cohort | Moderate | Moderate | Low | Low | Low | Moderate | Low |
| Fricker, 1994 | Prospective cohort | Moderate | Moderate | Low | Low | Low | Moderate | Low |
| Fricker, 1998 | Prospective cohort | Moderate | Moderate | Low | Low | Low | Moderate | Low |
| Miguel, 1995 | Prospective cohort | Moderate | Moderate | Low | Low | Low | Moderate | Low |
| Fowler, 1998 | Retrospective cohort | Moderate | Moderate | Low | Low | Low | Moderate | Low |
| Ireland, 2002 | Prospective cohort | Moderate | Moderate | Low | Low | Low | Moderate | Low |
| Romano, 2012 | Prospective cohort | Moderate | Moderate | Low | Low | Low | Moderate | Low |
| Pandis, 2006 | Prospective cohort | Moderate | Moderate | Low | Low | Low | Moderate | Low |
| Ozer, 2014 | Prospective cohort | Moderate | Moderate | Low | Low | Low | Moderate | Low |
