# Supplementary material for: Clinical bracket failure rates between different bonding techniques: a systematic review and meta-analysis
Source: Eur J Orthod. 2022 Oct 12;45(2):175–85. doi: 10.1093/ejo/cjac050 (PMC10065138; doi:10.1093/ejo/cjac050)
Supplement: cjac050_suppl_Supplementary_Table_S7 [file cjac050_suppl_supplementary_table_s7.docx]

**Question:** SEP compared to CM-AEP for bracket bonding

| **Certainty assessment** | | | | | | | **№ of patients** | | **Effect** | | **Certainty** | **Importance** |
| --- | --- | --- | --- | --- | --- | --- | --- | --- | --- | --- | --- | --- |
| **№ of studies** | **Study design** | **Risk of bias** | **Inconsistency** | **Indirectness** | **Imprecision** | **Other considerations** | **SEP** | **CMAEP** | **Relative (95% CI)** | **Absolute (95% CI)** |  |  |
| **Bracket failure at 6 months (follow-up: mean 6; assessed with: Number of failed brackets)** | | | | | | | | | | | | |
| 10 | observational studies | not serious | serious^a^ | not serious | not serious | none | 85/2389 (3.6%) | 86/2389 (3.6%) | **RR 1.04** (0.67 to 1.61) | **1 more per 1,000** (from 12 fewer to 22 more) | ⨁◯◯◯ Very low | IMPORTANT |
| **Bracket failure at 12 months (follow-up: mean 12; assessed with: Number of failed brackets)** | | | | | | | | | | | | |
| 8 | observational studies | not serious | serious^b^ | not serious | not serious | none | 151/3083 (4.9%) | 105/3057 (3.4%) | **RR 1.37** (0.98 to 1.92) | **13 more per 1,000** (from 1 fewer to 32 more) | ⨁◯◯◯ Very low | IMPORTANT |
| **Bracket failure at 18+ months (follow-up: mean 18; assessed with: Number of failed brackets)** | | | | | | | | | | | | |
| 5 | observational studies | not serious | not serious | not serious | not serious | none | 103/2123 (4.9%) | 110/2119 (5.2%) | **RR 0.93** (0.72 to 1.20) | **4 fewer per 1,000** (from 15 fewer to 10 more) | ⨁⨁◯◯ Low | IMPORTANT |

**CI:** confidence interval; **RR:** risk ratio

#### Explanations

a. I-squared=38.9%

b. I-squared=42.2%

**Question:** SCR compared to RM-GIC for bracket bonding

| **Certainty assessment** | | | | | | | **№ of patients** | | **Effect** | | **Certainty** | **Importance** |
| --- | --- | --- | --- | --- | --- | --- | --- | --- | --- | --- | --- | --- |
| **№ of studies** | **Study design** | **Risk of bias** | **Inconsistency** | **Indirectness** | **Imprecision** | **Other considerations** | **SCR** | **RMGIC** | **Relative (95% CI)** | **Absolute (95% CI)** |  |  |
| **Bracket failure at 12 months (follow-up: mean 12; assessed with: Number of failed brackets)** | | | | | | | | | | | | |
| 10 | observational studies | not serious | not serious | not serious | not serious | none | 172/3110 (5.5%) | 368/3188 (11.5%) | **RR 0.38** (0.24 to 0.61) | **72 fewer per 1,000** (from 88 fewer to 45 fewer) | ⨁⨁◯◯ Low | IMPORTANT |
| **Bracket failure at 18+ months (follow-up: mean 18; assessed with: Number of failed brackets)** | | | | | | | | | | | | |
| 4 | observational studies | not serious | very serious^a^ | not serious | not serious | none | 145/919 (15.8%) | 342/935 (36.6%) | **RR 0.44** (0.37 to 0.52) | **205 fewer per 1,000** (from 230 fewer to 176 fewer) | ⨁◯◯◯ Very low | IMPORTANT |

**CI:** confidence interval; **RR:** risk ratio

#### Explanations

a. I-squared=78.9%
