# Supplementary material for: Clinical bracket failure rates between different bonding techniques: a systematic review and meta-analysis
Source: Eur J Orthod. 2022 Oct 12;45(2):175–85. doi: 10.1093/ejo/cjac050 (PMC10065138; doi:10.1093/ejo/cjac050)
Supplement: cjac050_suppl_Supplementary_Data [file cjac050_suppl_supplementary_data.docx]

**Qualitative analysis**

In our meta-analysis we identified various contributing factors that may substantially affect the bonding failure of orthodontic brackets.

Fluoride varnish may weaken bonding forces in the case of the SEP bonding technique. Two independent clinical trials with a 6-month follow-up time concluded that the bracket failure rate is significantly higher after the use of a fluoride-containing varnish in comparison with pumice (1,2). Conversely, cleaning the dental surfaces with pumice decreases bond failure rates (3,4). After three months follow up, the omission of pumice before the application SEP resulted in significantly more (11.4%) bond failures compared to the pumice-based conventional protocol (2.4%) (3). In another similar study with six months follow-up, the failure rate was 32.2% in the pumice group and 55.6% at the pumice-free group (4).

Tooth whitening may also increase the bonding failure of brackets. Performing a 180-day long clinical trial, Mullins et al. (5) concluded that tooth bleaching with 38% hydrogen peroxide for 30 minutes significantly increased bond failures. While the failure rate in the unbleached group was only 1.8%, in the tooth whitening group the failure rate was 16.6%. In patients undergoing similar cures, a delay of 2-3 weeks before orthodontic treatment is recommended (5).

Studies prove that in the CM-AEP technique, various light sources are equally appropriate for curing the cement. Light sources with their recommended curing times, whether halogen light, extra-large halogen transmitter, plasma or LED units, do not significantly affect bond failure. (6–11). A significant difference was found in the work of Elaut el al. between argon (2.4%) and halogen (5.7%) sources of light (12). The reduction of chair time is an advantage of alternative curing light sources, whereas no significant differences in bond performance was detected between lamp types (6–11).

In the CM-AEP technique, omitting the primer did not significantly increase bracket failure, as Bazargani et al. found in a 24 month follow-up period when a primer was not used (5.5%) in comparison with those when a primer was used (3.1%) as suggested by the manufacturer (13). Another similar study concluded that bonding without a primer (15.8%) is statistically non-inferior to bonding with a primer (11.1%) when adhesive precoated brackets are used (14). Other studies investigated the effect of various primers on bracket failure. They found no significant differences in performance between various primers (15,16). Taken together, omitting the step of using primers can reduce the time of bracket cementing without increasing the risk of bracket failure.

Several studies showed that changing the etching time (15s, 30s, 60s) and concentration of phosphoric acid (2%, 15%, 37%) had no effect on the bond failures of self-cure composites (17–20). Additionally, sandblasting was shown not to affect the performance of metal brackets used with RM-GIC during a 20‑month observation period (21). The materials from which brackets are manufactured may also affect their survival rate. Hitmi et al. found significantly higher bond failures in resin brackets (8.4%) than metal (7.2%) or ceramic brackets (0.7%) in combination with RM-GIC (22).

Summarizing the above observations, there are several confounding factors that might influence bracket failure. These factors mostly originate from the modification of techniques in order to decrease bracket failure, to decrease chair time, or to make the teeth less susceptible to caries. Various light sources with equal power, etching time, and phosphoric acid concentration seem not to affect bonding failure (6–11,17–20). Therefore, chairside time could be decreased by using a high intensity light source to decrease both the need for exposure and the etching time, in combination with ceramic brackets for minimizing failure rate.

1. Talic NF. Effect of fluoridated paste on the failure rate of precoated brackets bonded with self-etching primer: A prospective split-mouth study. Am J Orthod Dentofac Orthop. 2011;140(4):527–30.

2. Grover S, Sidhu MS, Prabhakar M, Jena S, Soni S. Evaluation of fluoride varnish and its comparison with pumice prophylaxis using self-etching primer in orthodontic bonding - An in vivo study. Eur J Orthod. 2012;34(2):198–201.

3. Lill DJ, Lindauer SJ, Tüfekçi E, Shroff B. Importance of pumice prophylaxis for bonding with self-etch primer. Am J Orthod Dentofac Orthop. 2008;133:423–6.

4. Burgess AM, Sherriff M, Ireland AJ. Self-etching primers: Is prophylactic pumicing necessary? A randomized clinical trial. Angle Orthod. 2006;76(1):114–8.

5. Mullins JM, Kao EC, Martin CA, Gunel E, Ngan P. Tooth whitening effects on bracket bond strength in vivo. Angle Orthod. 2009;79(4):777–83.

6. Sfondrini MF, Cacciafesta V, Scribante A. Plasma arc versus halogen light curing of orthodontic brackets: A 12-month clinical study of bond failures. Am J Orthod Dentofac Orthop. 2004;1253:342–7.

7. Frost T, Norevall L-I, Persson M. Bond strength and clinical efficiency for two light guide sizes in orthodontic bracket bonding. Br J Orthod. 1997;24(1):35–40.

8. Koupis N, Eliades T, Athanasiou AE. Clinical evaluation of bracket bonding using two different polymerization sources. Angle Orthod. 2008;78(5):922–5.

9. Mirabella D, Spena R, Scognamiglio G, Luca L, Gracco A, Siciliani G. LED vs Halogen Light-Curing of Adhesive-Precoated Brackets. Angle Orthod. 2008;78(5):935–40.

10. Oz AA, Oz AZ, Arici S. In-vitro bond strengths and clinical failure rates of metal brackets bonded with different light-emitting diode units and curing times. Am J Orthod Dentofac Orthop. 2016;149(2):212–6.

11. Russell J, Littlewood S, Blance A, Mitchell L. The efficacy of a plasma arc light in orthodontic bonding: A randomized controlled clinical trial. J Orthod. 2008;35(3):202–9.

12. Elaut J, Wehrbein H. The effects of argon laser curing of a resin adhesive on bracket retention and enamel decalcification: a prospective clinical trial. Eur J Orthod. 2004;26(5):553–60.

13. Bazargani F, Magnuson A, Löthgren H, Kowalczyk A. Orthodontic bonding with and without primer: a randomized controlled trial. Eur J Orthod. 2016;38(5):503–7.

14. Nandhra SS, Littlewood SJ, Houghton N, Luther F, Prabhu J, Munyombwe T, et al. Do we need primer for orthodontic bonding? A randomized controlled trial. Eur J Orthod. 2015;37(2):147–55.

15. Varlik SK, Demirbaş E. Effect of light-cured filled sealant on the bond failure rate of orthodontic brackets in vivo. Am J Orthod Dentofac Orthop. 2009;135(2):144.e1-144.e4.

16. Wenger NA, Deacon S, Harradine NWT. A randomized control clinical trial investigating orthodontic bond failure rates when using Orthosolo universal bond enhancer compared to a conventional bonding primer. J Orthod. 2008;35(1):27–32.

17. Carstensen W. Clinical results after direct bonding of brackets using shorter etching times. Am J Orthod. 1986;89:70–2.

18. Carstensen W. Clinical effects of reduction of acid concentration on direct bonding of brackets. Angle Orthod. W. Carstensen; 1993;63(3):221–4.

19. Kinch AP, Taylor H, Warltler R, Oliver RG, Newcombe RG. A clinical trial comparing the failure rates of directly bonded brackets using etch times of 15 or 60 seconds. Am J Orthod Dentofac Orthop. 1988;94(6):476–83.

20. Sadowsky PL, Retief DH, Hernández-Orsini R, Rape WG, Bradley EL. Effects of etchant concentration and duration on the retention of orthodontic brackets: An in vivo study. Am J Orthod Dentofac Orthop. 1990;98:417–21.

21. Ozer M, Arici S. Sandblasted Metal Brackets Bonded with Resin-modified Glass Ionomer Cement In Vivo. Angle Orthod. 2005;75(3):406–9.

22. Hitmi L, Muller C, Mujajic M, Attal J-P. An 18-month clinical study of bond failures with resin-modified glass ionomer cement in orthodontic practice. Am J Orthod Dentofac Orthop. 2001;120(4):406–15.
